# Seaweed-Derived Halogenated Monoterpenes as Lead Compounds in Schistosomiasis Control

**DOI:** 10.3390/pharmaceutics18070767

**Published:** 2026-06-23

**Authors:** Sara Guibunda Tajú, Amanda Beatriz da Silva Soares, Patrícia Aoki Miyasato, Rafaela Paula de Freitas, Lenita de Freitas Tallarico, Erika Mattos Stein, Pio Colepicolo, Eliana Nakano

**Affiliations:** 1 Research Center in Biodiversity and Conservation—CPBC, Púnguè University, Chimoio 333, Manica, Mozambique; sarabibitaju@gmail.com; 2 Laboratory of Parasitology, Butantan Institute, São Paulo 05503-900, Brazil; amanda.ssoares@icb.usp.br (A.B.d.S.S.); patricia.aoki@fundacaobutantan.org.br (P.A.M.); rafaela.freitas@fundacaobutantan.org.br (R.P.d.F.); lenita.tallarico@fundacaobutantan.org.br (L.d.F.T.); 3 Laboratory of Algae Biochemistry and Molecular Biology, Institute of Chemistry, University of São Paulo, São Paulo 05508-090, Brazil; stein.erika.m@gmail.com (E.M.S.); piocolep@iq.usp.br (P.C.)

**Keywords:** *Schistosoma mansoni*, *Ochtodes secundiramea*, nuclear magnetic resonance—NMR, halogenated monoterpenes, ochtodenes

## Abstract

**Background/Objectives:** Schistosomiasis, a parasitic disease caused by *Schistosoma* worms with freshwater snails as intermediate hosts, affects over 250 million people. The current control relies solely on praziquantel, which raises concerns on drug resistance and highlights the need for new therapeutic alternatives. Our bioprospection studies have focused on marine macroalgae as an unexplored source of antischistosomal metabolites with promising results. Guided by WHO recommendations to target both the parasite and its transmission vectors, this study aimed to investigate *Ochtodes secundiramea* to: (i) isolate active metabolites; (ii) evaluate the isolated compounds against adult worms and oviposition to identify leads for drug development; and (iii) perform an independent screening of their effects against the environmental transmission stages on cercariae and *B. glabrata* embryos. **Methods:** A dichloromethane extract of *O. secundiramea* was submitted to an NMR–biomonitored guided fractionation against *Schistosoma mansoni* adult worms. Active fractions were further purified through HPLC and characterized by ^1^H and ^13^C NMR spectroscopy to identify the isolated compounds. **Results:** Three halogenated monoterpenes were isolated: ochtodene **1** (4-bromo-1,6,8-trichloro-2,3-ochtodene), ochtodene **2** (2-chloro-1,6,8-tribromo-3,8-ochtodene), and the novel natural product ochtodene **3** [2,6-dibromo-4-(2-chloroethylidene)-1,1dimethylcyclohexane]. Ochtodene **1** was the primary active metabolite against *Schistosoma mansoni* adult worms, with IC_50_/96 h values of 47.2 and 46.1 µM for male and female worms respectively, and totally suppressed egg laying with 60 µM, while showing no toxicity toward human fibroblasts. Notably, all metabolites, including the novel ochtodene **3**, caused 100% mortality in cercariae and embryos at low concentrations. **Conclusions:** The discovery of the novel ochtodene **3** and the identification of distinct leads for host treatment and transmission elimination position *O. secundiramea* as a promising source for integrated schistosomiasis control.

## 1. Introduction

Schistosomiasis is a parasitic disease affecting over 240 million people worldwide [1], with the second highest per capita burden among all Neglected Tropical Diseases [2]. Caused by trematode worms of the genus *Schistosoma*, the infection is acquired through penetration of larval forms of the parasite—released by freshwater snails—during contact with infested water [3]. Praziquantel has been the only recommended drug for the treatment and control of the disease for more than 40 years, raising concerns on drug resistance; still, its large-scale administration to millions of people through preventive chemotherapy highlights the urgent need for new therapeutic alternatives. On another front, aiming to interrupt schistosomiasis transmission, the search for snail control products—preferably cost-effective and with low risk to humans and the environment—is being encouraged by the WHO [4], in line with the Global Vector Control Response (GVCR) goal to reduce the incidence of vector-borne diseases, including schistosomiasis, by at least 40% by 2025 [5].

Our bioprospection studies have focused on marine macroalgae as an alternative source of active compounds of potential use in schistosomiasis control with promising results. In a comprehensive screening with 37 Brazilian seaweed species for molluscicidal activity against *Biomphalaria glabrata* embryos and anthelminthic activity against *Schistosoma mansoni*, 22 species (60%) were active in at least one of the two models [6].

Unlike the extensively explored plant species, the antiparasitic effects of seaweed metabolites have been poorly studied [7]. Nonetheless, marine macroalgae may contribute with novel scaffolds to the library of bioactive compounds. Halogenated metabolites, for example, while rare in terrestrial species, are common in marine species due to the abundance of chloride and bromide ions in seawater [8,9,10,11]. Our results with *Laurencia dendroidea* [12], where three halogenated sesquiterpenes were responsible for the antischistosomal and molluscicidal activities, suggest that halogenated terpenes may be good leads to the development of drugs and products for schistosomiasis treatment and control.

While our previous screenings identified the antischistosomal potential of *Ochtodes* extracts [6], the specific metabolites responsible for these activities remained unknown. This study fills this gap by performing the first bioguided isolation and comparative evaluation of its major halogenated monoterpenes. Therefore, *Ochtodes secundiramea* was submitted to a combined NMR–biomonitored guided fractionation protocol to identify and isolate its active metabolites. First, the isolated compounds were evaluated against adult worms and oviposition to identify leads for drug development. Second, to assess their potential for environmental transmission control, the compounds were independently tested against *S. mansoni* cercariae and *B. glabrata* embryos.

## 2. Materials and Methods

### 2.1. Algae Sampling

*Ochtodes secundiramea* samples were collected in Espírito Santo State, Southeastern Brazil, and vouchers of representative specimens were deposited in the Herbário da Universidade de São Paulo under SPF: 58241 and SPF: 58242. After collection, the fresh algae were washed thoroughly with seawater to remove sand particles and epiphytes. The cleaned material was stored frozen in zip-lock plastic bags at −20 °C until preparation of the extracts.

### 2.2. Obtention of Extract and Chromatography Analyses

After lyophilization and grinding, extracts were obtained by maceration of the material with dichloromethane 1:10 (*m/v*). The resultant extract was centrifuged at 10,000 rpm for 10 min, filtered, and concentrated under reduced pressure. This procedure was repeated three times.

The final extract dichloromethane was analyzed employing thin-layer chromatography (TLC) and gas chromatography–mass spectrometry (GC-MS).

Thin-layer chromatography was performed on normal-phase silica plates (20 cm × 10 cm × 0.1 mm) with a UV indicator (254 nm). The plates were then derivatized with Komarowsky reagent (mixture of 1 mL of 50% sulfuric acid with 10 mL of p-hydroxybenzaldehyde in ethanol (2%)) followed by heating [13].

Approximately 1.7 mg of each extract was prepared at a concentration of 1 mg/mL in chloroform. Samples were transferred to vials and analyzed using a gas chromatograph coupled to a quadrupole mass spectrometer (GC–MS-QP2010 Plus, Shimadzu, Kyoto, Japan) equipped with an HP-5MS capillary column ((5–phenyl)-dimethylpolysiloxane stationary phase; 30 m × 250 µm × 0.25 µm).

Chromatographic conditions were as follows: Injection temperature was 220 °C; oven temperature was increased at a rate of 3 °C/min from 60 to 240 °C, and held for 40 min. The interface and ion source (detector) temperatures were maintained at 240 °C. The injection volume was 1 µL in split mode (split ratio 1:5). Helium was used as the carrier gas at a constant flow rate of 1 mL/min. Ionization was performed by electron impact (EI) with collision energy of 70 eV. Mass spectra were acquired in full scan mode over the *m*/*z* range 40–1000, using the NIST 08 and NIST 08s libraries available in the GCMS-QP2010 Plus system (Shimadzu), as well as the NIST/EPA/NIH Mass Spectral Library (Version 2.0) and the NIST Mass Spectral Search Program (Nist08 and Nist08s).

Compound identification was achieved by comparing the mass spectra obtained for each peak with those suggested by the instrument libraries and by calculating the Kovats retention index (KI), followed by comparison with literature data. The KI values were calculated based on the retention times (Rts) of a homologous series of n-alkane standards (C8–C20 and C21–C40; Sigma-Aldrich, St. Louis, MO, USA). For each detected compound, library matches were considered according to the similarity index provided by the software. A similarity index cut-off of ≥85% was established for KI calculation and compound assignment.

### 2.3. Biomonitored Fractioning

Fractionation of the dichloromethane extract was carried out by liquid column chromatography (LCC) under ambient pressure using a glass column (1.20 m × 3 cm) packed with silica gel 60 (0.063–0.200 mm; 70–230 mesh ASTM; Merck, Darmstadt, Germany). Elution was performed using a gradient system of petroleum ether/acetone (85:15, 60:40, and 1:1, *v*/*v*) to achieve improved fractionation. Finally, 100% methanol was applied to ensure complete elution of the extract.

The chemical profiles (fingerprints) of the collected fractions were monitored by thin-layer chromatography (TLC) (description in Section 2.2), and fractions exhibiting similar chromatographic patterns were pooled. The resulting pooled fractions were subsequently analyzed by GC–MS and ^1^H NMR and evaluated for schistosomicidal activity (see Section 2.5).

### 2.4. Purification and Characterization of Ochtodenes

Subfractionation was performed using a preparative HPLC system (Shimadzu) equipped with an autosampler. An injection volume of 100 µL of the sample solution (1 g/mL) was introduced onto a silica column (Shim-pack PREP; 20 mm i.d.; 5 µm particle size, Merck KGaA, Darmstadt, Germany).

Elution was carried out under gradient conditions using petroleum ether and acetone. The gradient program started at 95% petroleum ether, which was gradually decreased to 40% over 25 min. The 40% petroleum ether composition was maintained for 5 min, followed by a gradual return to 95% petroleum ether over 20 min. The flow rate was set at 3 mL/min. Fractions were collected at 1 min intervals using an automated fraction collector; however, the collection time was manually adjusted when necessary to ensure that individual chromatographic peaks were collected in the same test tube. Detection was performed using a diode array detector (DAD) with ultraviolet absorption monitored over the wavelength range of 190–800 nm.

The chemical structures of the purified compounds were elucidated using spectroscopic and spectrometric methods. The 1D (^1^H and ^13^C) and 2D (COSY, HSQC and HMBC) NMR spectra were recorded on a Bruker Avance III 500 MHz spectrometer (Billerica, MA, USA) equipped with a 5 mm TXI probe with dedicated channels for ^1^H, ^13^C, and ^15^N, inverse detection, and a field-gradient system. ^13^C spectra were acquired at 125 MHz and ^1^H spectra at 500 MHz. Samples were dissolved in CDCl_3_, and spectral data were processed and analyzed using Bruker TopSpin 3.0 software.

In addition, mass spectrometry (MS) analyses were conducted for structural determination of the isolated compounds.

### 2.5. Schistosomicidal Activity in Adult Worms

The life cycle of *S. mansoni* (Sambon, 1907) (Trematoda: Schistosomatidae) (BH strain—Belo Horizonte, MG, Brazil) was maintained in *Biomphalaria glabrata* (Say, 1818) (Gastropoda: Planorbidae) snails and *Mesocricetus auratus* (Waterhouse, 1839) (Mammalia: Cricetidae) hamsters.

The in vitro activity assay was performed according to a protocol established in our laboratory as previously described [6,12,14]. Briefly, adult worms were recovered through portal perfusion from hamsters 42 days after infection. Five coupled male and female worms were exposed to the test compounds; praziquantel was used as the positive control and dimethylsulfoxide (DMSO) as the negative control. Worms were maintained in 24-well culture plates at 37 °C and 5% CO_2_, and monitored after 2 h and then every 24 h thereafter for 96 h for motility, morphological alterations, and reproduction.

### 2.6. Evaluation of Cytotoxicity in Healthy Cells

#### MTT Cytotoxicity Test

The human fibroblast cells used in this study were kindly provided by Silvya Stuchi Maria-Engler (FCF-USP, São Paulo, Brazil). The cytotoxicity was determined by the colorimetric MTT (tetrazolium) assay [15]. Briefly, cells were incubated for 24 h in 96-well plates at 1 × 10^4^ cells/mL. Control cells and/or cells treated with 12.5, 25, 50, 250, 500 and 1000 μM of ochtodene **1** were incubated at 37 C in a 5% CO_2_ atmosphere. After 24 h, the supernatant was removed and MTT 0.5% in DMEM was added. After 4 h, supernatant was removed and 100 μL of DMSO was added to dissolve the intracellular dark-blue formazan crystal formed. After a few minutes at room temperature, absorbance was read at 570 nm.

### 2.7. Molluscicidal Activity

Groups of egg masses, with at least 50 embryos each, were placed in 12-well culture plates and kept at 25 ± 2 °C. The organisms were exposed to solutions of ochtodenes (50, 25, 12.5 and 6.25 µg/mL) for 24 h and then transferred to another plate containing dechlorinated water for seven days. Dechlorinated water/DMSO (2%) was used as the negative control. Organisms were evaluated daily, employing a stereoscope microscope, for lethality or teratogenic effects. All tests were conducted in triplicate.

### 2.8. Cercaricidal Activity

The cercaricidal activity was determined according to an adapted protocol from dos Santos et al. 2022 [12]. *S. mansoni* cercariae were obtained by exposing infected *B. glabrata* snails to artificial light for 2 h to stimulate larval shedding. A suspension containing ~100 cercariae/mL in dechlorinated water was distributed to a 24-well culture microplate (1 mL/well) and exposed to a solution of ochtodenes (50 µg/mL); DMSO 2% in dechlorinated water was used as a negative control. Cercarial viability was assessed under a stereomicroscope observation at 5, 15, 30, 60, and 120 min post-exposure. The assays were performed in triplicate and mortality was determined by observing complete loss of motility and tail detachment.

### 2.9. Statistical Analysis

Lethality data for *Schistosoma mansoni* adult worms were used to calculate lethal concentrations (LC_50_ and LC_90_) and 95% confidence intervals (CIs) via Probit Analysis [16]. The graphs were prepared using Microsoft Excel Version 26 H2 (Windows 11 Pro, Microsoft Corp, Redmond, DC, USA).

## 3. Results and Discussion

### 3.1. Isolation of Ochtodenes from NMR and Bioguided Fractioning of Ochtodes secundiramea (Montagne) M. Howe 1920

*Ochtodes secundiramea* was one of the selected species in our previous trial studies with crude extracts of macroalgae for antischistosomal and molluscicidal activities [6], where *O. secundiramea* chloroform extract induced 100% mortality in both male and female worms. Females were slightly more sensitive than male worms to the chloroform extract. Reproduction was totally inhibited, with 100% couple separation and complete inhibition of oviposition. Tested for molluscicidal activity on *Biomphalaria glabrata* embryos at blastulae and veliger stages, *O. secundiramea* extract induced 100% of mortality on embryos at both stages [6].

In the present study, DCM extract of *Ochtodes secundiramea* was submitted to a combined NMR/biomonitored guided fractioning in *Schistosoma mansoni* worms. A dichloromethane extract of *O. secundiramea* was fractionated to generate 292 fractions, which were grouped into 38 fractions after thin-layer chromatography fingerprinting and tested at 100 µg/mL on adult worms. Seventeen out of the 38 fractions showed some level of lethality. Fractions inducing 100% of mortality and inhibiting the oviposition were re-fractioned through HPLC and tested at 50 µg/mL. The subfractions were analyzed by ^1^H RMN, and those with clean proton spectra were submitted to ^13^C RMN. Those fractions with impurities in the ^1^H RMN spectrum were grouped according to profile similarities and re-fractioned to isolate the active compound. 2D NMR techniques COSY, HSQC and HMBC along with GC-MS were employed to characterize the isolated compounds.

**Figure 1:**
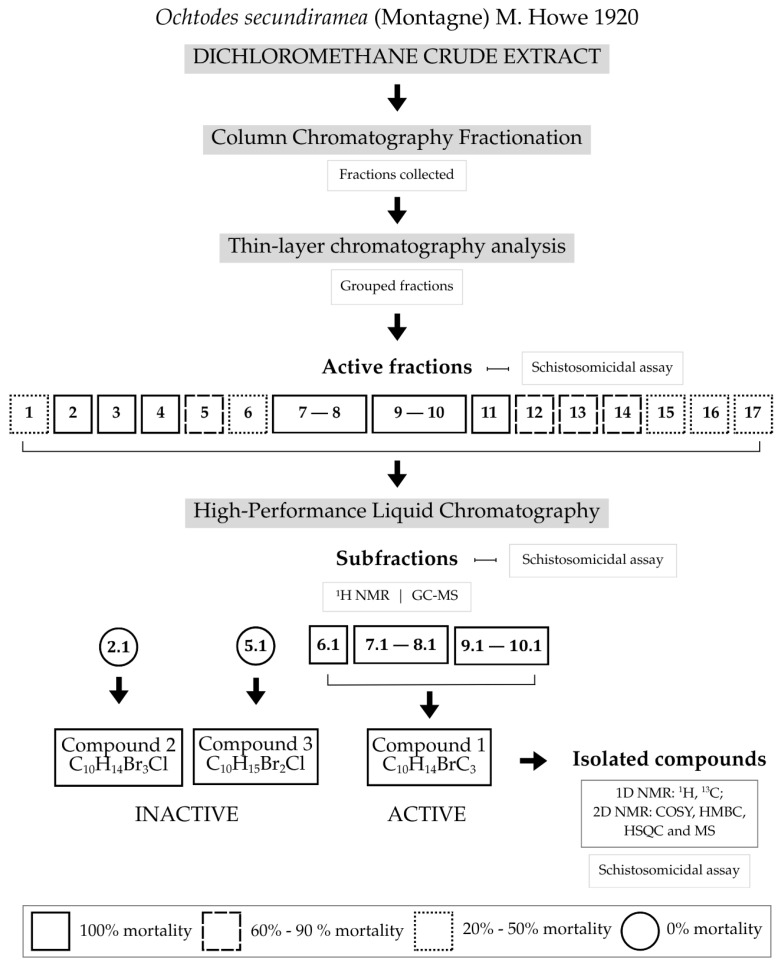
Biomonitored fractionation of active extract from marine macroalgae *Ochtodes secundiramea* (Montagne) M. Howe 1920. CCF—column chromatography fractionation; NMR—nuclear magnetic resonance; GC-MS—gas chromatography–mass spectrometry; COSY—correlation spectroscopy, HMBC—heteronuclear multiple-bond correlation; HSQC—heteronuclear single-quantum coherence.

**Figure 2:**
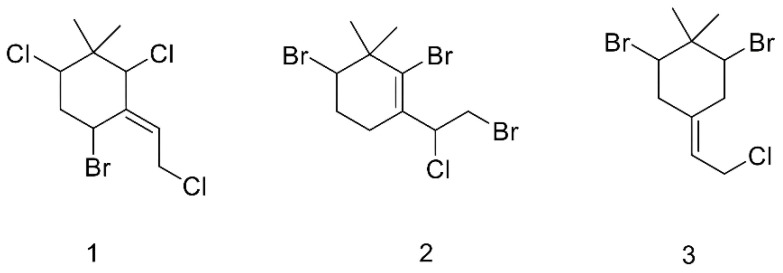
Ochtodenes isolated from marine macroalgae *Ochtodes secundiramea* (Montagne) M. Howe 1920. GC-MS: ochtodene **1** *m*/*z* 320, MM: 320.48 Da; ochtodene **2** *m*/*z* 410, MM: 409.383 Da; ochtodene **3** *m*/*z* 331, MM: 330.48 Da.

The main constituents of the active fractions were isolated and identified as ochtodene **1**, ochtodene **2** and ochtodene **3** (Figure 1 and Figure 2).

Ochtodene **1** (C_10_H_14_BrCl_3_; 4-bromo-1,6,8-trichloro-2-3-ochtodene) was first isolated from the red macroalgae *Portieria hornemannii* (Gigartinales) originating from the Philippines by [17] and later from cultured *O. secundiramea* (Gigartinales) extracts by [10] aiming to obtaining halogenated monoterpenes.

Ochtodene **2** (C_10_H_14_Br_3_Cl; 2-chloro-1,6,8-tribromo-3-8-ochtodene) was first identified by Gerwick from *O. secundiramea* [11] and showed no activity when tested for antibacterial and antitumoral activities. No antitumoral activity was also reported by [17] for ochtodene **2** isolated from *P. hornemanni*.

This is the first report for ochtodene **3** (C_10_H_15_Br_2_Cl). The structure of the new compound was elucidated based on detailed analysis of its ^1^H and ^13^C NMR spectroscopic data, which were consistent with a polyhalogenated monoterpene of the ochtodene type. The ^13^C NMR spectrum displayed ten carbon signals, in agreement with a C_10_ skeleton, comprising two methyl groups, three methylenes, three methines, and two quaternary carbons, as established by HSQC analysis.

The presence of an olefinic system was indicated by signals at δC 127.3 (CH-2) and 134.7 (C-3), with the corresponding proton resonance at δH 5.94 (t), confirming a trisubstituted double bond.

Several deshielded aliphatic carbons at δC 56.2 (CH_2_-1), 60.0 (CH-5), and 60.2 (CH-7), with corresponding proton signals at δH 4.13 (t), 5.09 (t), and 4.85 (1H, dd, H-7), are characteristic of carbons bearing halogen substituents, supporting the presence of bromine and/or chlorine atoms in the molecule.

Additional methylene signals at δC 32.0 (CH_2_-4) and 34.6 (CH_2_-8), with proton resonances at δH 3.78 (2H, ddd) and 2.75 (2H, m), further define the saturated portion of the cyclic framework.

Two methyl singlets at δH 1.20 (s) and 1.30 (s) correlate with δC 19.7 and 28.8, respectively. And the quaternary carbons at δC 134.7 (C-3) and 40.3 (C-6) complete the carbon framework. Altogether, these data are in close agreement with those reported for ochtodene derivatives by McConnell and Fenical [18], supporting the assignment of a polyhalogenated cyclic monoterpene structure.

The EI mass spectrum exhibited a molecular ion cluster at *m*/*z* 327/329/331/333, displaying the characteristic isotopic pattern of a molecule containing Br_2_Cl. Prominent fragment ions at *m*/*z* 249 (M–Br) and 213 (M–Br–Cl) reflect sequential loss of halogen atoms, a typical fragmentation behavior of polyhalogenated monoterpenes. Additional ions at *m*/*z* 167, 131, and 91 arise from rearranged hydrocarbon fragments following dehalogenation, supporting a substituted cyclohexene framework. Together, these data corroborate the proposed molecular formula C_10_H_15_Br_2_Cl and the assigned ochtodene-type structure (Table 1 and Table S1).

The ^13^C and ^1^H-NMR data for **1**–**3** are provided in Table 1.

Among the macroalgae metabolites tested for biological activities, halogenated monoterpenes are one of the most active [10]. Found primarily in only three genera of red macroalgae, *Plocamium*, *Portieria*, and *Ochtodes* [19], the production of halogenated monoterpenes by marine species has been associated with chemical defense against herbivory [20].

Although *Plocamium* and *Portieria* are recognized as the main sources of halogenated monoterpenes (HMTs) [20], *Ochtodes* has also been demonstrated as a significant source of HMTs [10,21]. In fact, in our trial studies, crude extracts of *Plocamium brasiliense* were active against *S. mansoni* only with 500 µg/mL but not with 100 µg/mL [14], in contrast with *Ochtodes secundiramea* extract, which killed 100% of the parasites at 100 µg/mL [6].

### 3.2. Biomonitored Fraction of Ochtodes secundiramea (Montagne) M. Howe 1920 Extract Against Schistosoma mansoni Led to Ochtodene **1**

The three ochtodenes isolated after bioguided fractioning of the DCM extract of *O. secundiramea* were tested for schistosomicidal activity at five concentrations ranging from 30 to 120 µM in five pairs of adult worms. Lethal effects were observed only for ochtodene **1**, which induced 100% mortality in all dose groups except for those exposed to the lowest concentration. After 24 h of exposure, ochtodene **1** killed 100% of the worms with 150, 120 and 90 µM; with 60 µM the compound killed 70% of the parasites after 96 h. An inhibition effect on the oviposition was observed at all tested concentrations; above 60 µM, ochtodene **1** completely inhibited the oviposition. Ochtodene **2** induced minor effects on motility at concentrations higher than 60 µM and affected the reproduction, inducing separation of four of the five couples and completely inhibiting the oviposition. Ochtodene **3** showed no effects on motility or reproduction (Table 2).

Whereas the small number of isolated ochtodenes limits a comprehensive SAR analysis, the comparative biological evaluation of the three isolated monoterpenes provides preliminary but significant insights into their Structure–Activity Relationship (SAR). While ochtodene **1** exhibited potent anthelmintic activity against *S. mansoni* adult worms, its close analogs **2** and **3** were found to be inactive in the same model. Structurally, these compounds differ primarily in their halogenation patterns and stereochemistry. In ochtodene **1**, the specific spatial orientation of the bromine and chlorine atoms appears to be a critical pharmacophore for interacting with essential parasite targets. The loss of activity observed in ochtodene **2** and **3** suggests that even minor structural modifications—such as changes in the chiral centers or the repositioning of the halogen atoms—disrupt the molecular recognition required for anthelmintic efficacy. This high structural specificity indicates that ochtodene **1** is a specialized lead scaffold, whereas the broader activity of all three compounds against cercariae and *B. glabrata* embryos suggests a different, perhaps more generalized, mechanism of action for environmental stages. While these initial observations do not constitute a full Structure–Activity Relationship (SAR) study, they highlight ochtodene **1** as a promising scaffold for future lead optimization.

De la Mare et al. [22] studied a series of halogenated monoterpenes with an identical monoterpene backbone but varying halogen substitutions, observing that more chlorine atoms led to higher anticancer activity, corroborating our findings where ochtodene **1**, with the highest number of chlorines, was the only monoterpene active against *Schistosoma* worms.

The potential of halogenated monoterpenes as bioactive compounds has already been reported in studies on anticancer, antiplasmodial, molluscicidal, and insecticidal activities with red macroalgae [17,21,22,23]. Nonetheless, the role of halogen moieties in monoterpene molecules is not well understood and warrants further studies with compound libraries expanded by systematic chemical substitutions [22].

**Figure 3:**
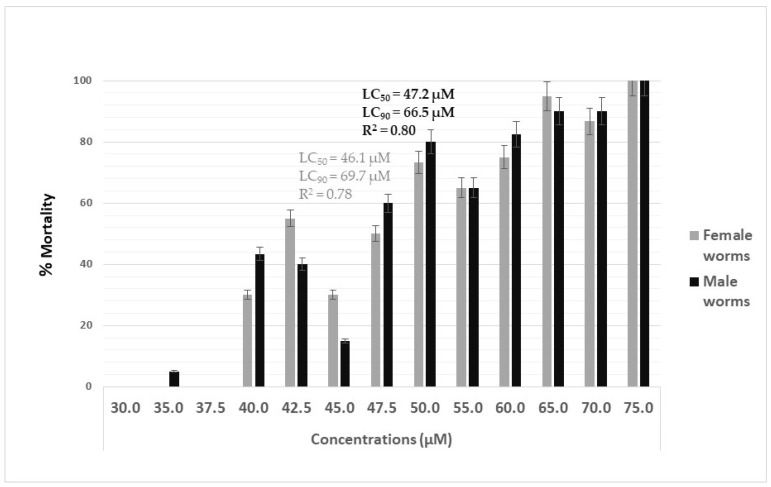
Concentration–response relationship after 96 h of exposure to ochtodene **1** in adult *Schistosoma mansoni* adult worms. Female = grey columns and male = black columns. Controls: Positive—100% mortality (praziquantel 1.5 μg/mL) and negative—non-active (RPMI media with DMSO 1.5% μg/mL). Endpoint observed was mortality, with the LC_50_, LC_90_, and R^2^ values indicated in the graph.

Ochtodene **1** was, therefore, chosen to be further investigated for antischistosomal activity. The compound was tested at 13 concentrations ranging from 30 to 75 µM in 10 paired worms per dose group (Table 3). Male worms were slightly more sensitive than the females to the compound. We used IC_50/96h_ values of 47.2 µM (15,13 µg/mL) and IC_90/96h_ of 66.5 μM (21.31 µg/mL) for male worms, and IC_50/96h_ values of 46.1 µM (14.77 µg/mL) and IC_90/96h_ of 69.7 μM (22.34 µg/mL) for female worms (Figure 3). Most notably, ochtodene **1** caused separation on 100% of the coupled worms and a complete suppression of egg laying at a concentration of 60 µM. This total inhibition of oviposition is a critical finding, as eggs are the primary cause of both the clinical pathology (granulomas) and the environmental transmission of schistosomiasis.

**Figure 4:**
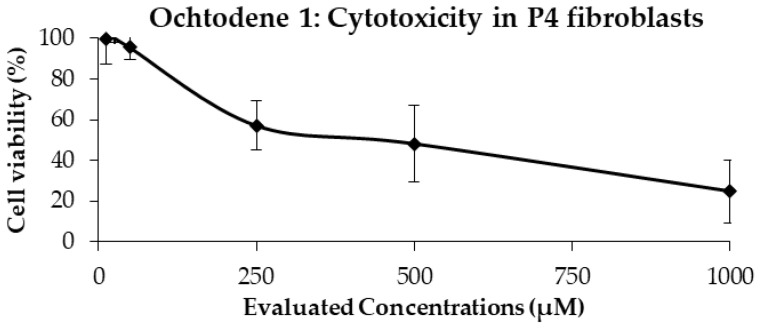
Viability of P4 fibroblast cells by MTT test within 24 h of exposure to ochtodene **1**.

The metabolic-activity-based MTT assay was employed to assess the cytotoxicity of ochtodene **1** on human fibroblasts (Figure 4). The compound exhibited low toxicity, with an IC_50_ of 450 µM. At a high concentration of 1000 µM, cell viability was reduced to 25%, whereas concentrations of 12.5, 25, and 50 µM maintained viability above 95%. A selectivity index (SI) close to 10 at this early discovery stage is a promising indicator of selective anthelmintic action, particularly when considering the biological differences between a cell monolayer and a multicellular parasite. While fibroblasts in culture are directly and uniformly exposed to the compound, adult schistosomes possess a complex tegument and robust physiological defenses. The fact that ochtodene **1** achieves 100% inhibition of egg laying at concentrations significantly below its cytotoxic threshold reinforces its potential as a lead candidate for further optimization.

Given the significant activity of ochtodene 1 against adult worms and its low toxicity toward human cells, and the moluscicidal activity of *O. secundiramea* crude extract [6], we subsequently investigated whether these halogenated monoterpenes could also interfere with the parasite’s life cycle outside the human host. Therefore, ochtodenes **1**–**3** were evaluated for their potential as environmental agents, targeting both the intermediate host and the free-swimming infective stages.

### 3.3. Ochtodenes for Environmental Control of Schistosomiasis

Ochtodenes **1**–**3** were screened against *B. glabrata* embryos and *S. mansoni* free-swimming cercariae to evaluate their potential as environmental agents for transmission control.

All three compounds demonstrated significant molluscicidal activity against blastula-stage embryos. At concentrations of 6.25–50 µg/mL, compounds **1** and **3** induced 100% lethality across the entire range, whereas 2 showed a dose-dependent effect, reaching 54% lethality at 50 µg/mL (Table 4).

Similarly to that observed in *S. mansoni* adult worms, a reduction in the effect after slight modifications to the chemical structure was observed for *B. glabrata* embryos, suggesting specificity for the molluscicidal activity of ochtodenes.

A clear dose-related effect on mortality was observed for ochtodene **2**, but it was not possible for ochtodene **1** and **3**, as all the exposed groups induced 100% of mortality and concentrations lower than 6.25 µg/mL were not tested. Therefore, differences in the activity of ochtodene **1** and **3** could not be verified. Nevertheless, the dose–response effect for ochtodene **2** suggests an intrinsic molluscicidal activity, one of the main objectives of laboratory testing of molluscides according to the WHO Guidelines [24].

In the present work, as in our previous studies [6,12], the molluscicidal activity was assessed in intracapsular embryos. Among the advantages of this model, *B. glabrata* embryos are very sensitive at low concentrations and require only small amounts of samples. Background levels of death and malformations are low and the effects are reproducible [25].

In addition, since the environmental control of schistosomiasis targeting cercariae has been proposed isolated or in association to snail control [12,26,27,28,29], we tested the three ochtodenes for activity against *S. mansoni* cercariae. Larvae were directly exposed to a single concentration of 50 µg/mL of each compound and assessed for the effects in motility. All three compounds induced total loss of motility in 100% of cercariae after 120 min of exposure. The immobilization effect was gradual for ochtodene **2**, but reached 100% after 5 min for ochtodenes **1** and **3** (Table 5). Further studies with additional concentrations are necessary to characterize the cercaricidal activity of the ochtodenes.

## 4. Conclusions

In conclusion, this study provides the first definitive chemical and biological characterization of the active monoterpenes from the red alga *Ochtodes secundiramea* against *Schistosoma mansoni*. The bioguided fractionation allowed the isolation of three halogenated monoterpenes, including the novel ochtodene **3**. Regarding drug development, ochtodene **1** presented activity against adult worms and completely suppressed oviposition at sub-lethal concentrations. As for environmental control, the isolated compounds showed independent activity against *S. mansoni* cercariae and *B. glabrata* embryos. By distinguishing between therapeutic action against adult worms and environmental intervention against transmission stages, these findings support distinct, complementary strategies for integrated schistosomiasis control.

## 5. Patents

Tajú, S. G.; Stein, E. M.; Nakano, E.; Colepicolo, P. Uso de compostos Octodenos Halogenados na preparação de um medicamento para prevenção e/ou tratamento de esquistossomose e para aplicação em águas infestadas por caramujos e cercárias. UNIVERSIDADE DE SÃO PAULO—USP. BR, (21) BR 10 2022 020701-1 A2, 11 October 2022. Available in: https://auspin.usp.br/ (accessed on 16 June 2026).

## Figures and Tables

**Table 1:** 1H and 13C-NMR assignments for compounds **1**–**3**.

Ochtodenes									
	1 (C_10_H_14_BrCl_3_)			2 (C_10_H_14_Br_3_Cl)			3 (C_10_H_15_Br_2_Cl)		
		δ^13^C (ppm)	δ^1^H (ppm, J in Hz)		δ^13^C (ppm)	δ^1^H (ppm, J in Hz)		δ^13^C (ppm)	δ^1^H (ppm, J in Hz)
1	CH_2_	37.6	4.19 (dd, 12.3, 9.4) 4.06 (dd, 12.3, 6.6)	CH_2_	31.9	3.65 (dd, 10.2, 6.1) 3.58 (dd, 10.2)	CH_2_	56.2	4.13 (t)
2	CH	131.8	5.98 (dd, 9.52, 6.58)	CH	62.0	5.45 (dd, 10.0, 6.1)	CH	127.3	5.94 (t)
3	C	137.8	-	C	131.0	-	C	134.7	-
4	CH	50.4	4.99 (d)	CH_2_	23.9	2.42 (m)	CH_2_	32.0	3.78 (ddd)
5	CH_2_	41.3	2.70 (dd, 4.9, 1.8) 2.68 (ddd, 5.7, 2.45)	CH_2_	28.5	2.32 (m)	CH	60.0	5.09 (t)
6	CH	52.7	4.85 (dd, 12.8, 4.3)	CH	61.1	4.33 (dd, 8.4, 2.9)	C	40.3	-
7	C	41.4	-	C	44.1	-	CH	60.2	4.85 (dd)
8	CH	69.9	4.40 (d)	C	134.5	-	CH_2_	34.6	2.75 (m)
9	CH_3_	28.5	1.03 (s)	CH_3_	27.4	1.37 (s)	CH_3_	19.7	1.2 (s)
10	CH_3_	20.5	1.30 (s)	CH_3_	28.4	1.55 (s)	CH_3_	28.8	1.3 (s)

**Table 2:** Activity of ochtodenes on *Schistosoma mansoni* adult worms.

**Ochtodenes**	**Mortality**			**Reproduction**		**Motility**
1 C_10_H_14_BrCl_3_	**Female**	**Male**	**Total**	**Couple** **Separation**	**Eggs (Average)**	
150 µM	+++	+++	+++	-	0	+++
120 µM	+++	+++	+++	-	0	+++
90 µM	+++	+++	+++	++	0	+++
60 µM	++	++	++	+++	0	+++
30 µM	-	-	-	++	29.9	+++
2 C_10_H_14_Br_3_Cl						
150 µM	-	-	-	+++	0	+
120 µM	-	-	-	++	0	+
90 µM	-	-	-	+	31.4	+
60 µM	-	-	-	-	84.2	-
30 µM	-	-	-	-	115	-
3 C_10_H_15_Br_2_Cl						
150 µM	-	-	-	-	55.8	-
120 µM	-	-	-	-	67.2	-
90 µM	-	-	-	-	90.4	-
60 µM	-	-	-	-	92	-
30 µM	-	-	-	-	89.2	-
Controls						
Positive *	+++	+++	+++	-	-	+++
Negative **	-	-	-	-	154	-

Legend: * Praziquantel 1.5 μg/mL; ** RPMI media with DMSO 1.5% μg/mL. Mortality: Activity: (+++) = 100% mortality; (++) = between 50% and 100%; (-) = non-active. Motility: (+++) = 100% reduction; (+) = slight reduction; (-) = no effect. Reproduction: (+++) = 100% separation; (++) = between 50% and 100%; (+) ≤50% separation; (-) = no effect.

**Table 3:** Activity of ochtodene **1** on *Schistosoma mansoni* adult worms.

Ochtodene 1 (C_10_H_14_BrCl_3_)	Mortality			Reproduction		Motility
	Female	Male	Total	Couple Separation	Eggs (Average)	
75 µM	+++	+++	+++	+++	0	+++
70 µM	++	++	++	+++	0	+++
65 µM	++	++	++	+++	0	+++
60 µM	++	++	++	+++	1	+++
55 µM	++	++	++	+++	1.6	+++
50 µM	++	++	++	+++	3.1	+++
47.5 µM	++	++	++	+++	4.8	+++
45 µM	+	+	+	+++	5.2	+++
42.5 µM	+	+	+	+++	6.8	+++
40 µM	+	+	+	+++	19.8	+++
37.5 µM	-	-	-	++	20.9	+
35 µM	-	-	-	++	21.2	+
30 µM	-	-	-	-	30.5	-
Controls						
Positive *	+++	+++	+++	-	0	+++
Negative **	-	-	-	-	304.7	-

Legend: * Praziquantel 1.5 μg/mL; ** RPMI media with DMSO 1.5% μg/mL. Mortality: Activity: (+++) = 100% mortality; (++) = between 50% and 100%; (+) ≤50% mortality; (-) = non-active. Motility: (+++) = 100% reduction; (+) = slight reduction; (-) = no effect. Reproduction: (+++) = 100% separation; (++) = between 50% and 100%; (-) = no effect.

**Table 4:** Activity of ochtodenes on *Biomphalaria glabrata* embryos at blastulae.

Concentration (µg/mL)	Ochtodene 1 (C_10_H_14_BrCl_3_)		Ochtodene 2 (C_10_H_14_Br_3_Cl)		Ochtodene 3 (C_10_H_15_Br_2_Cl)	
	N° Embryos	Mortality	N° Embryos	Mortality	N° Embryos	Mortality
50 µg/mL	59	+++	52	++	68	+++
25 µg/mL	50	+++	55	+	52	+++
12.5 µg/mL	51	+++	66	-	64	+++
6.25 µg/mL	60	+++	54	-	53	+++
Controls						
Negative *	53	-	64	-	50	-
Negative **	51	-	55	-	58	-

Legend: * Dechlorinated water; ** dechlorinated water with DMSO 2% μg/mL. Activity: (+++) = 100% mortality; (++) = between 50% and 100%; (+) ≤50% mortality; (-) = non-active.

**Table 5:** Activity of ochtodenes against cercariae of *Schistosoma mansoni*.

Ochtodene	Total Cercariae	Mortality (%)				
		5 (min)	15 (min)	30 (min)	60 (min)	120 (min)
1 (C_10_H_14_BrC_l3_)	156	+++	+++	+++	+++	+++
2 (C_10_H_14_Br_3_Cl)	158	+	+	++	++	+++
3 (C_10_H_15_Br_2_Cl)	170	+++	+++	+++	+++	+++
Controls						
Negative *	143	-	-	-	-	+
Negative **	135	-	-	-	+	+

Legend: * Dechlorinated water; ** dechlorinated water with DMSO 2% μg/mL. (+++) 100% of cercariae motionless at the bottom of the test plate, (++) between 50% and 100% of cercariae motionless at the bottom of the test plate, (+) between 10% and 50% of cercariae motionless at the bottom of the test plate, and (-) lack of larvicidal activity with ≥90% of larvae swimming.

## Data Availability

The original contributions presented in this study are included in the article/Supplementary Materials. Further inquiries can be directed to the corresponding author.

## Supplementary Materials

The following supporting information can be downloaded at https://www.mdpi.com/article/10.3390/pharmaceutics18070767/s1, Table S1: Activity of fractions of the dichloromethane extract of *Ochtodes secumdiramea* (Montagne) M. Howe 1920 on *Schistosoma mansoni* adult worms; Figure S1. ^13^C NMR spectrum of ochtodene **1**; Figure S2: ^1^H NMR spectrum of ochtodene **1**; Figure S3: HSQC correlation at ochtodene **1**; Figure S4: HMBC correlation at ochtodene **1**; Figure S5: COSY correlation in ochtodene **1**; Figure S6: Mass spectrum of ochtodene **1**; Figure S7: ^13^C NMR spectrum of ochtodene **2**; Figure S8: ^1^H NMR spectrum of ochtodene **2**; Figure S9: HSQC correlation at ochtodene **2**; Figure S10: COSY correlation in ochtodene **2**; Figure S11: HMBC correlation at ochtodene **2**; Figure S12: Mass spectrum of ochtodene **2**; Figure S13: ^13^C NMR spectrum of ochtodene **3**; Figure S14: ^1^H NMR spectrum of ochtodene **3**; Figure S15: HSQC correlation at ochtodene **3**; Figure S16: HMBC correlation at ochtodene **3**; Figure S17: COSY correlation in ochtodene **3**; Figure S18: Mass spectrum of ochtodene **3**.

## References

[1] World Health Organization (WHO). https://www.who.int/health-topics/schistosomiasis#tab=tab_1.

[2] Li Q., Li Y., Guo S., Li S., Wang Q., Lin W., Zhang L., Li S., Zhou X., Xu J. (2025). Global trends of schistosomiasis burden from 1990 to 2021 across 204 countries and territories: Findings from GBD 2021 study. Acta Trop..

[3] Gryseels B., Polman K., Clerinx J., Kestens L. (2006). Human schistosomiasis. Lancet.

[4] Ntuli D.M.M., World Health Organization (WHO) (2020). Control of Neglected Tropical Diseases (NTD).

[5] World Health Organization (WHO) (2017). Global Vector Control Response 2017–2030.

[6] Stein E.M., Tajú S.G., Miyasato P.A., de Freitas R.P., Tallarico L.F., Dos Santos G.S., Luiz G.L.F., Rofatto H.K., da Silva F.N.V., Colepicolo P. (2021). The Prospective Use of Brazilian Marine Macroalgae in Schistosomiasis Control. Mar. Drugs.

[7] Falkenberg M., Nakano E., Zambotti-Villela L., Zatelli G.A., Philippus A.C., Imamura K.B., Velasquez A.M.A., Freitas R.P., Tallarico L.D.F., Colepicolo P. (2018). Bioactive compounds against neglected diseases isolated from macroalgae: A review. J. Appl. Phycol..

[8] Gribble G.W. (2024). A Survey of Recently Discovered Naturally Occurring Organohalogen Compounds. J. Nat. Prod..

[9] Naylor S., Hanke F.J., Manes L.V., Crews P. (1983). Chemical and Biological Aspects of Marine Monoterpenes. Fortschritte der Chemie Organischer Naturstoffe/Progress in the Chemistry of Organic Natural Products.

[10] Polzin J.J., Rorrer G.L., Cheney D.P. (2003). Metabolic flux analysis of halogenated monoterpene biosynthesis in microplantlets of the macrophytic red alga *Ochtodes secundiramea*. Biomol. Eng..

[11] Gerwick W.H. (1984). 2-chloro-1,6(S*),8-tribromo-3-(8)(Z)-ochtodene: A metabolite of the tropical red seaweed *Ochtodes secundiramea*. Phytochemistry.

[12] dos Santos G.S., Miyasato P.A., Stein E.M., Colepicolo P., Wright A.D., Pereira C.A.B., Falkenberg M., Nakano E. (2022). Algal-Derived Halogenated Sesquiterpenes from *Laurencia dendroidea* as Lead Compounds in Schistosomiasis Environmental Control. Mar. Drugs.

[13] Waksmundzka-Hajnos M., Sherma J., Kowalska T. (2008). Thin Layer Chromatography in Phytochemistry.

[14] Stein E.M., Machado L.P., Roffato H.K., Miyasato P.A., Nakano E., Colepicolo P., Andreguetti D.X. (2015). Antischistosomal activity from Brazilian marine algae. Rev. Bras. Farmacogn..

[15] Basch P.F. (1981). Cultivation of Schistosoma mansoni in vitro. I. Establishment of cultures from cercariae and development until pairing. J. Parasitol..

[16] Finney D.J. (1952). Probit Analysis (2nd ed.). J. Inst. Actuar..

[17] Fuller R.W., Cardellina J.H., Kato Y., Brinen L.S., Clardy J., Snader K.M., Boyd M.R. (1992). A pentahalogenated monoterpene from the red alga *Portieria hornemannii* produces a novel cytotoxicity profile against a diverse panel of human tumor cell lines. J. Med. Chem..

[18] McConnell O.J., Fenical W. (1978). Ochtodene and ochtodiol: Novel polyhalogenated cyclic monoter-penes from the red seaweed *Ochtodes secundiramea*. J. Org. Chem..

[19] Cikoš A., Jurin M., Čož-Rakovac R., Gašo-Sokač D., Jokić S., Jerković I. (2021). Update on sesquiterpenes from red macroalgae of the *Laurencia* genus and their biological activities (2015–2020). Algal Res..

[20] Paul V.J., Hay M.E., Duffy J.E., Fenical W., Gustafson K. (1988). Chemical defense in the seaweed *Ochtodes secundiramea* (Montagne) Howe (Rhodophyta): Effects of its monoterpenoid components upon diverse coral-reef herbivores. J. Exp. Mar. Biol. Ecol..

[21] König G.M., Wright A.D., Sticher O. (1990). A new polyhalogenated monoterpene from the red alga *Plocamium cartilagineum*. J. Nat. Prod..

[22] de la Mare J.A., Lawson J.C., Chiwakata M.T., Beukes D.R., Edkins A.L., Blatch G.L. (2012). Quinones and halogenated monoterpenes of algal origin show anti-proliferative effects against breast cancer cells in vitro. Investig. New Drugs.

[23] Fuller R.W., Cardellina J.H., Jurek J., Scheuer P.J., Alvarado-Lindner B., McGuire M., Gray G.N., Steiner J.R., Clardy J., Menez E. (1994). Isolation and structure/activity features of halomon-related antitumor monoterpenes from the red alga *Portieria hornemannii*. J. Med. Chem..

[24] Ali S.M., Allan F., Ayi I., Chandre F., Coelho P.M.Z., El-Hawary A.K., Jiamrong D., Kariuki C., N’Goran E.K., Madsen H. (2017). Field Use of Molluscicides in Schistosomiasis Control Programmes: An Operational Manual for Programmemanagers.

[25] de Freitas Tallarico L., Borrely S.I., Hamada N., Grazeffe V.S., Ohlweiler F.P., Okazaki K., Granatelli A.T., Pereira I.W., Pereira C.A., Nakano E. (2014). Developmental toxicity, acute toxicity and mutagenicity testing in freshwater snails *Biomphalaria glabrata* (Mollusca: Gastropoda) exposed to chromium and water samples. Ecotoxicol. Environ. Saf..

[26] Valente R., Diaz J.I., Salomón O.D., Navone G.T. (2016). The role of *Phyllocaulis variegatus* (Mollusca: Veronicellidae) in the transmission of digenean parasites. Rev. Mex. Biodivers..

[27] Acheampong D.O., Owusu-Adzorah N., Armah F.A., Aninagyei E., Asiamah E.A., Thomford A.K., Anyan W.K. (2020). Ethnopharmacological evaluation of schistosomicidal and cercaricidal activities of some selected medicinal plants from Ghana. Trop. Med. Health.

[28] Kamel B., Laidemitt M.R., Lu L., Babbitt C., Weinbaum O.L., Mkoji G.M., Loker E.S. (2021). Detecting and identifying *Schistosoma* infections in snails and aquatic habitats: A systematic review. PLoS Negl. Trop. Dis..

[29] Medina J.M., Peixoto J.L.B., Silva A.A., Haraguchi S.K., Falavigna D.L.M., Zamuner M.L.M., Sarragiotto M.H., Vidotti G.J. (2009). Evaluation of the molluscicidal and *Schistosoma mansoni* cercariae activity of Croton floribundus extracts and kaurenoic acid. Rev. Bras. Farm..

