# Supplementary material for: Seaweed-Derived Halogenated Monoterpenes as Lead Compounds in Schistosomiasis Control
Source: Pharmaceutics. 2026 Jun 23;18(7):767. doi: 10.3390/pharmaceutics18070767 (PMC13414796; doi:10.3390/pharmaceutics18070767)
Supplement: Supplementary file 1 [file pharmaceutics-18-00767-s001.zip › pharmaceutics-4323370-supplementary.pdf]

Seaweed-derived halogenated monoterpenes as lead compounds in schistosomiasis control

Sara Guibunda Tajú [1,2], Amanda Beatriz da Silva Soares [2,3], Patrícia Aoki Miyasato [2], Rafaela Paula de Freitas [2], Lenita de Freitas Tallarico [2], Erika Mattos Stein [3], Pio Colepicolo [3], and Eliana Nakano [2,*]

Table S1 - Activity of fractions of the dichloromethane extract of *Ochtodes secumdiramea* (Montagne) M. Howe 1920 on *Schistosoma mansoni* adult worms.

	Mortality (%)			
	**Female**	**Male**	**Total**	**Eggs (average)**
**Crude extract**				
Dichlorometane	100	100	100	0
**Fractions**				
**1**	0	0	0	124
**2**	0	0	0	120
**3**	0	0	0	157
**4**	0	0	0	118.4
**5**	60	0	30	5
**6**	0	0	0	77.8
**7**	100	100	100	0
**8**	100	100	100	0
**9**	100	100	100	0
**10**	80	60	70	0
**11**	80	20	50	0
**12**	0	0	0	0
**13**	100	100	100	0
**14**	100	100	100	0
**15**	100	100	100	0
**16**	100	100	100	0
**17**	100	100	100	0
**18**	20	100	60	3
**19**	0	0	0	80
**20**	0	0	0	151.2
**21**	80	100	90	2
**22**	0	0	0	118.4
**23**	0	0	0	160.4
**24**	0	0	0	44.2
**25**	0	0	0	83.4
**26**	0	0	0	81
**27**	100	20	60	40.6
**28**	20	20	20	94.2
**29**	0	0	0	158.8
**30**	0	0	0	99

**31**	20	80	50	43.6
**32**	0	0	0	170.2
**33**	0	0	0	127.4
**34**	80	0	40	12
**35**	0	0	0	31.4
**36**	0	0	0	50.6
**37**	0	0	0	109.4
**38**	0	0	0	116.6
Controls				
**Positive ***	100	100	100	0
**Negative****	0	0	0	170.2

Legend: *Praziquantel 1.5 µg/mL; **RPMI media with DMSO 1.5% µg/mL.

Chromatogram of *O. secundiramea* extract

The chromatogram shows the presence of octodene 1 (peak 60, RT = 42.21 min), octodene 2 (peak 70, RT = 45.24 min), and octodene 3 (peak 80, RT = 47.80 min).

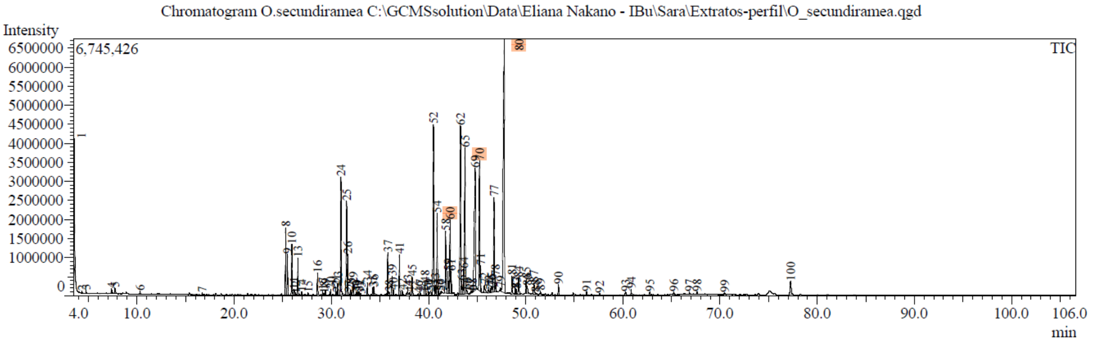

Ochtodene 1:

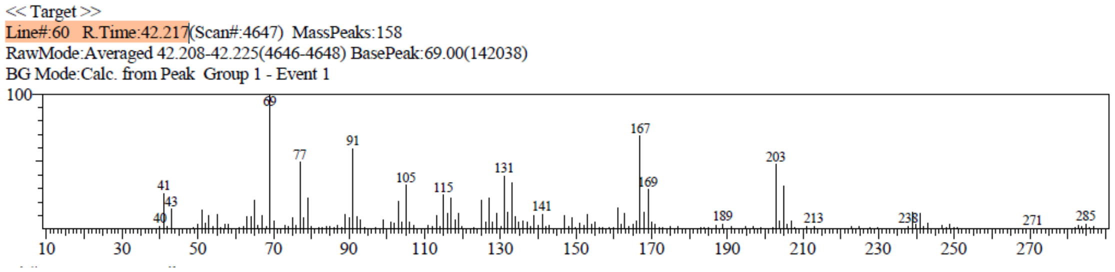

Ochtodene 2:

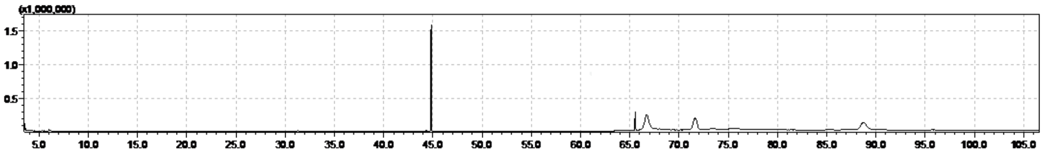

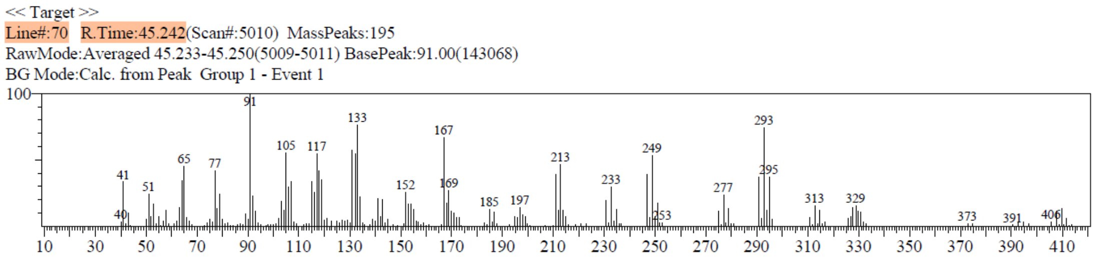

Ochtodene 3:

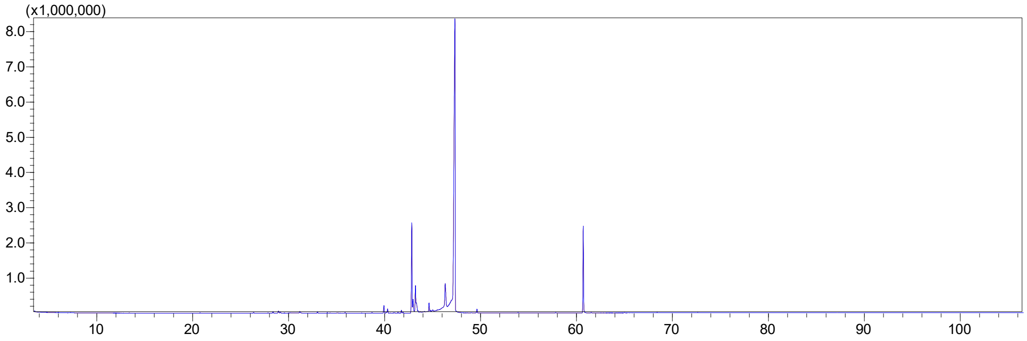

<< Target >>
Line#:80 R.Time:47.800(Scan#:5317) MassPeaks:182
RawMode:Averaged 47.792-47.808(5316-5318) BasePeak:166.95(451922)
BG Mode:Calc. from Peak Group 1 - Event 1

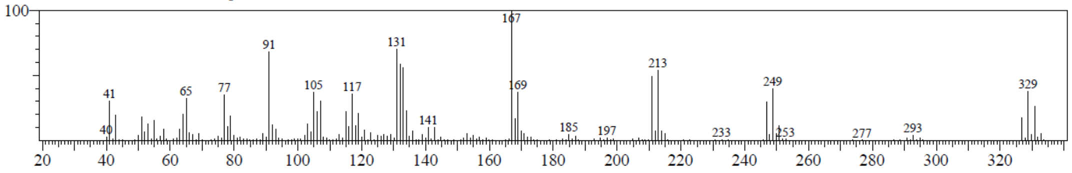

Ochtodene 1 ($C_{10}H_{14}BrC_{13}$):

[13]Carbon NMR spectrum (CDC_{13}, 500 MHz)

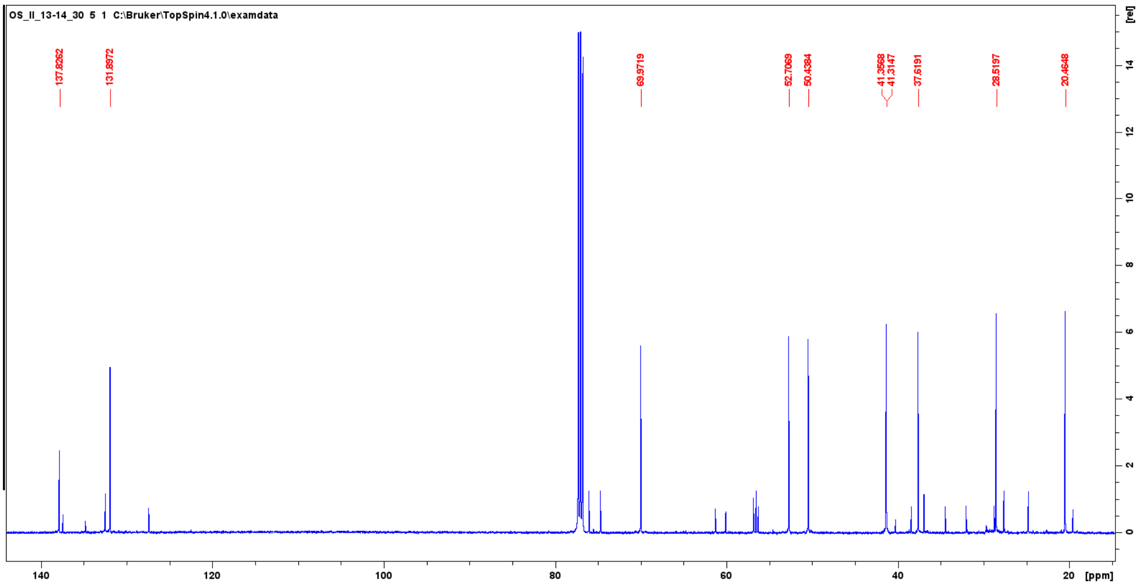

Figure S1. ^{13}C NMR spectrum of ochtodene 1.

^{1}H NMR spectrum ($CDCl_3$, 500 MHz)

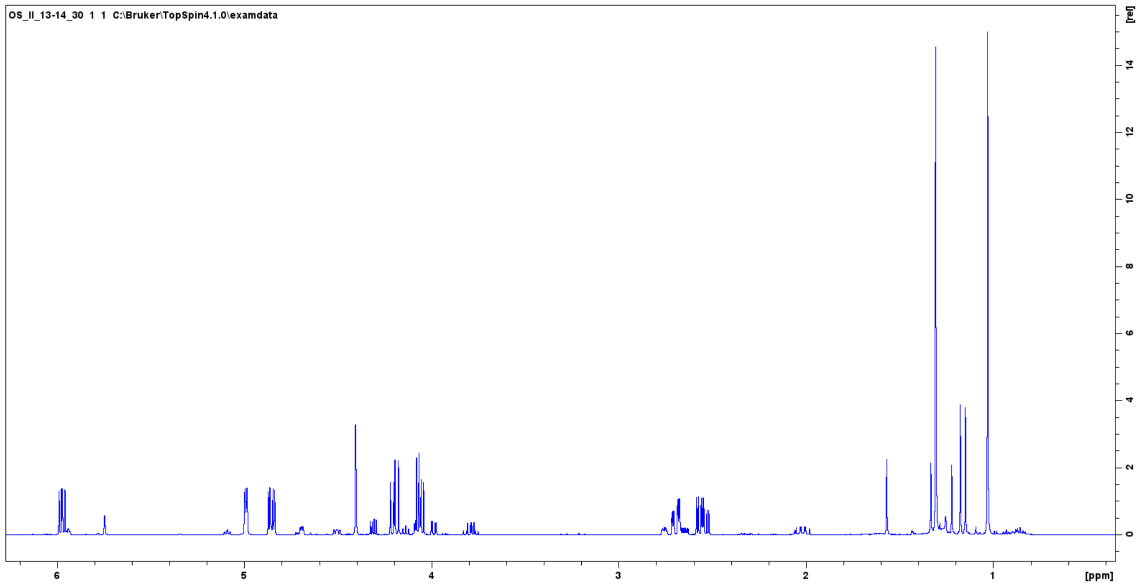

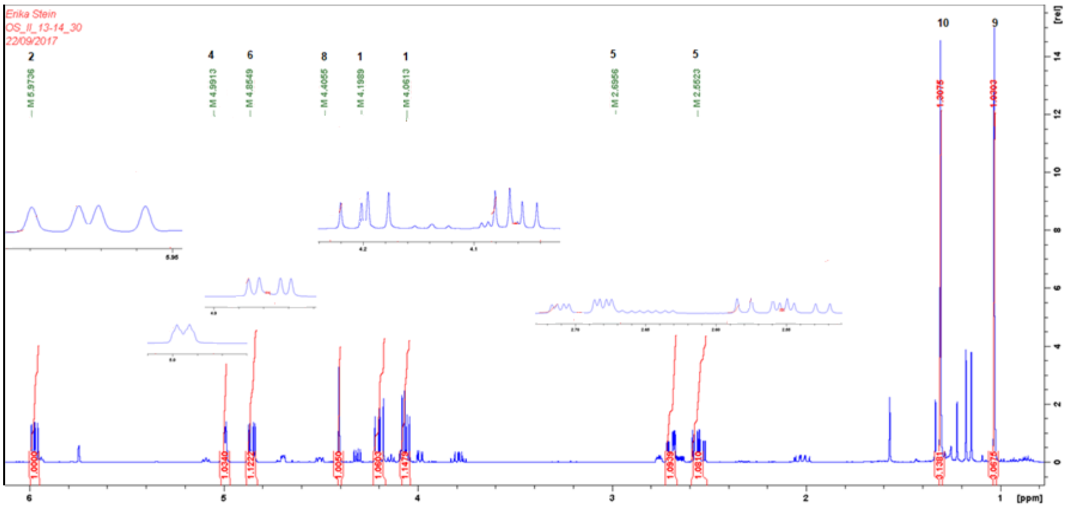

Figure S2. ^{1}H NMR spectrum of ochtodene 1.

Heteronuclear Single Quantum Correlation – HSQC ($CDCl_3$, 500 MHz)

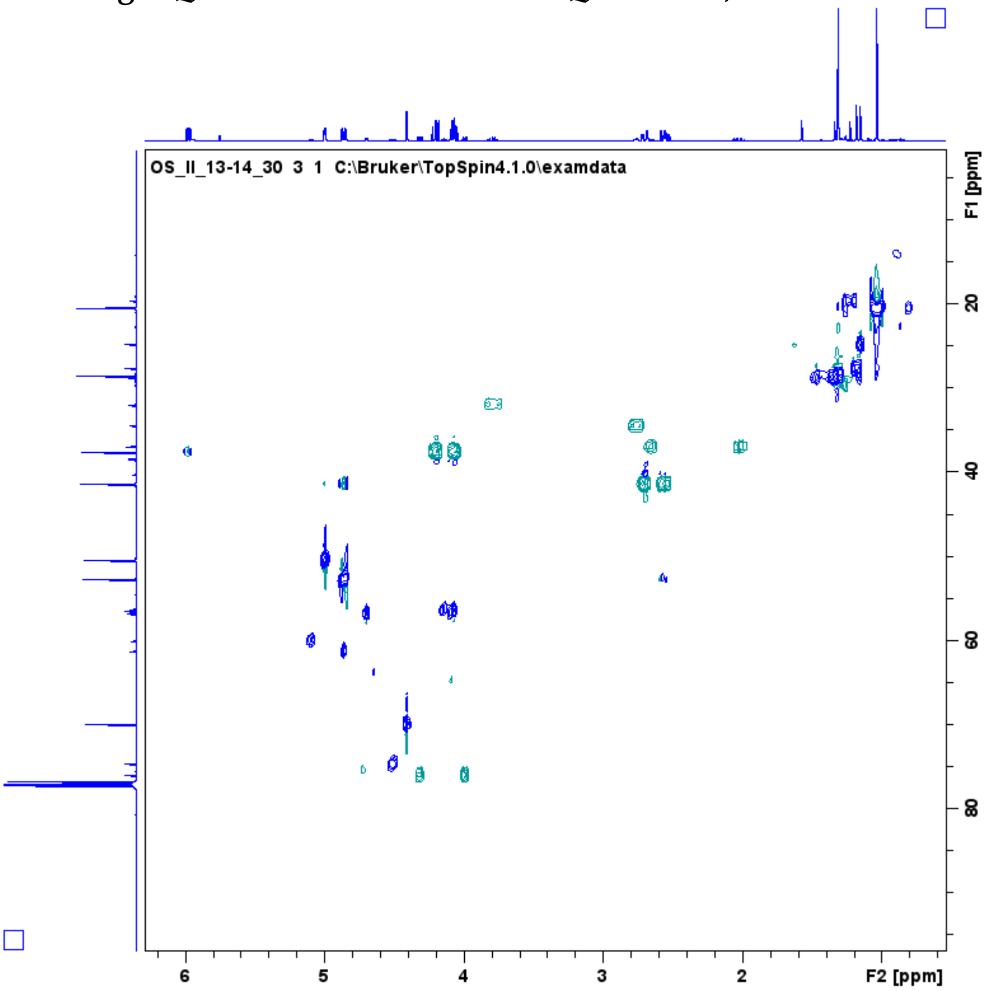

Figure S3. HSQC correlation at ochtodene 1.

Heteronuclear Multiple Bond Correlation – HMBC (CDCl3, 500 MHz)

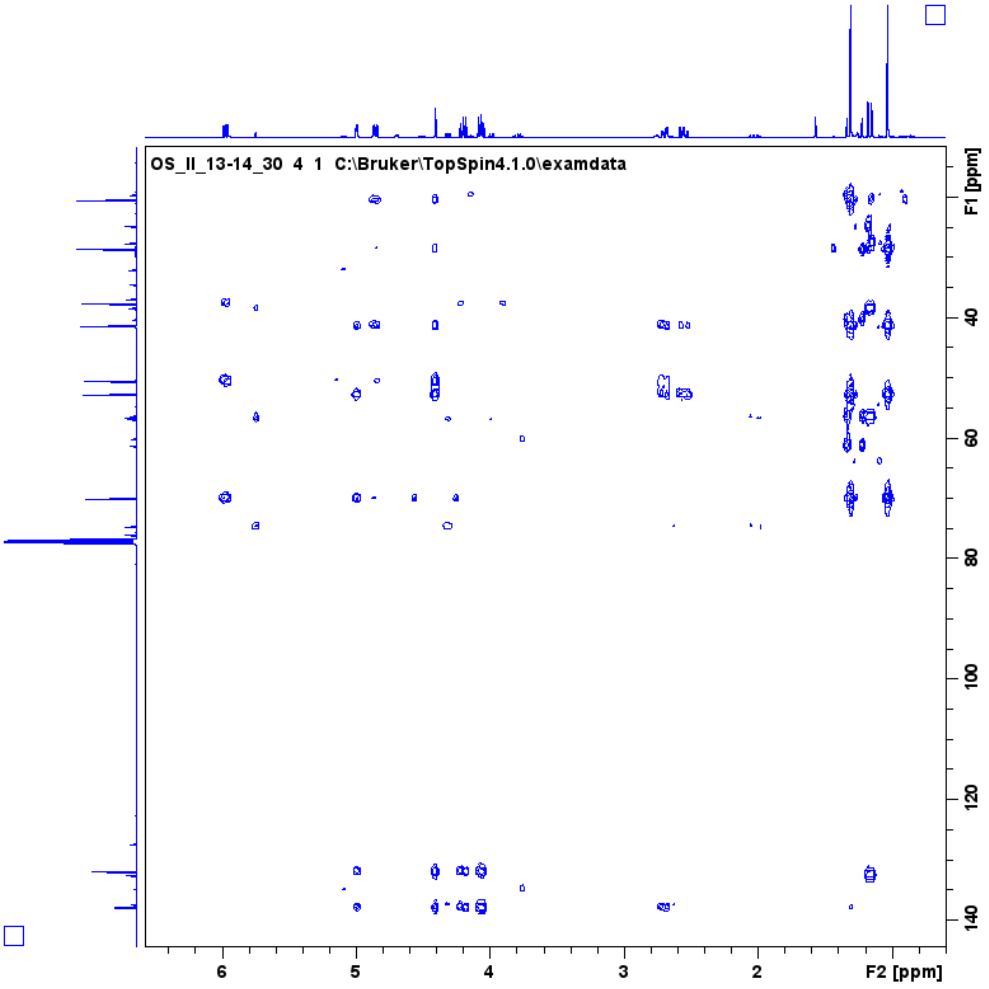

Figure S4. HMBC correlation at ochtodene 1.

Correlation Spectroscopy – COSY (CDCl3, 500 MHz)

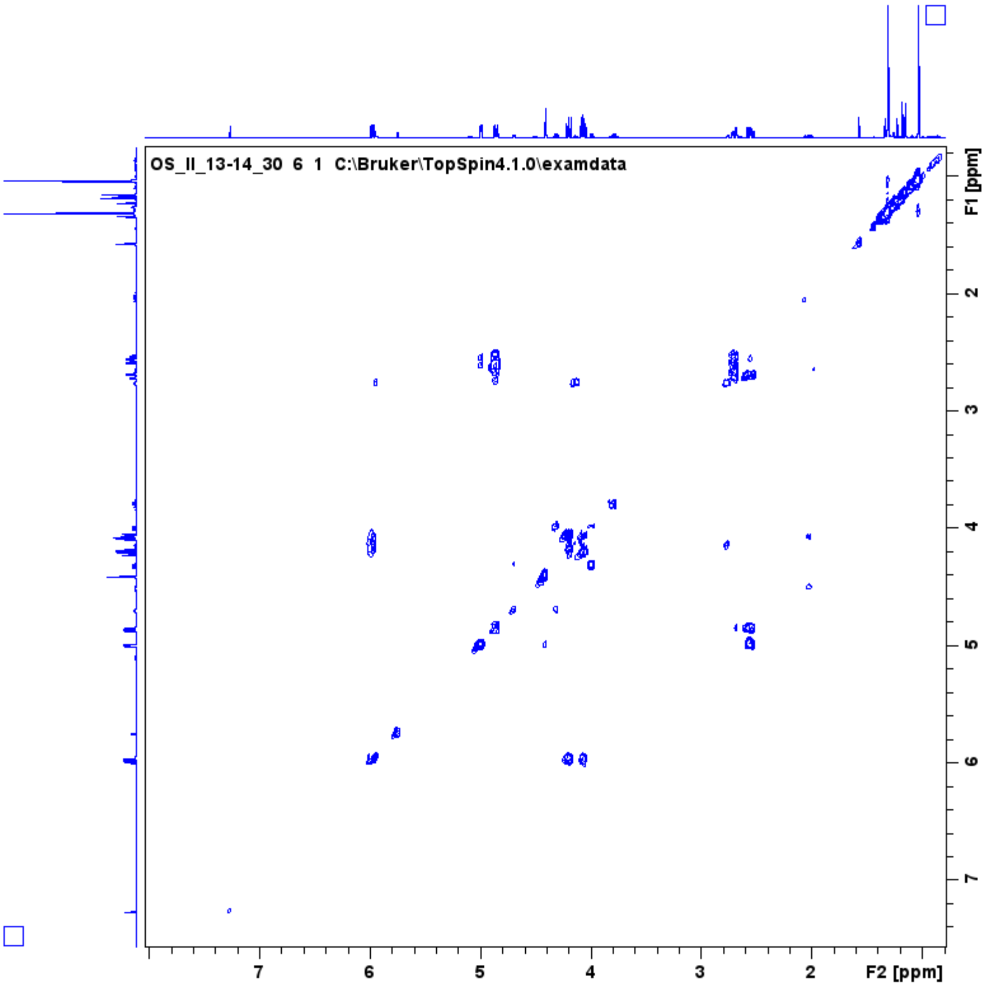

Figure S5. COSY correlation in ochtodene 1.

Mass Spectrometry - MS

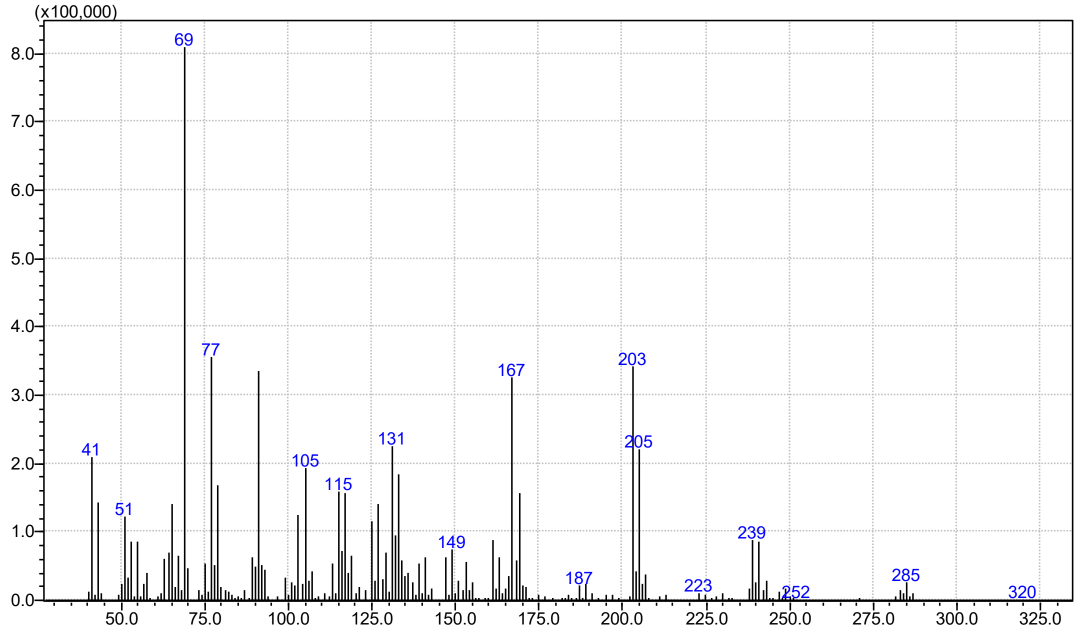

Figure S6. Mass spectrum of ochtodene 1.

Ochtodene 2 ($C_{10}H_{14}Br_3Cl$):

[13]Carbom NMR spectrum (CDCl3, 500 MHz)

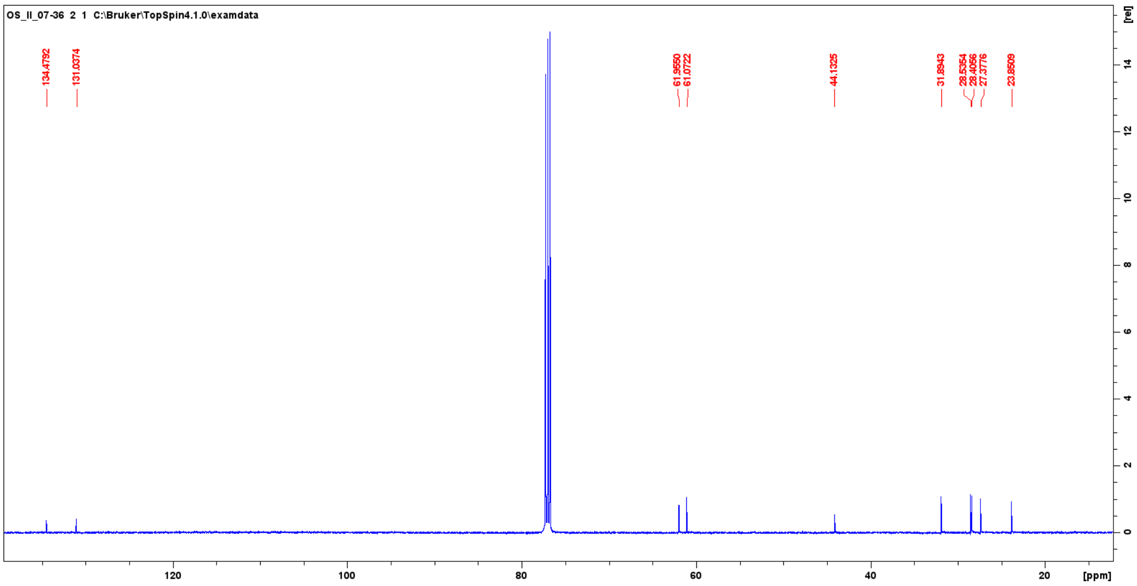

Figure S7. ^{13}C NMR spectrum of ochtodene 2.

^{1}H NMR spectrum ($CDCl_3$, 500 MHz)

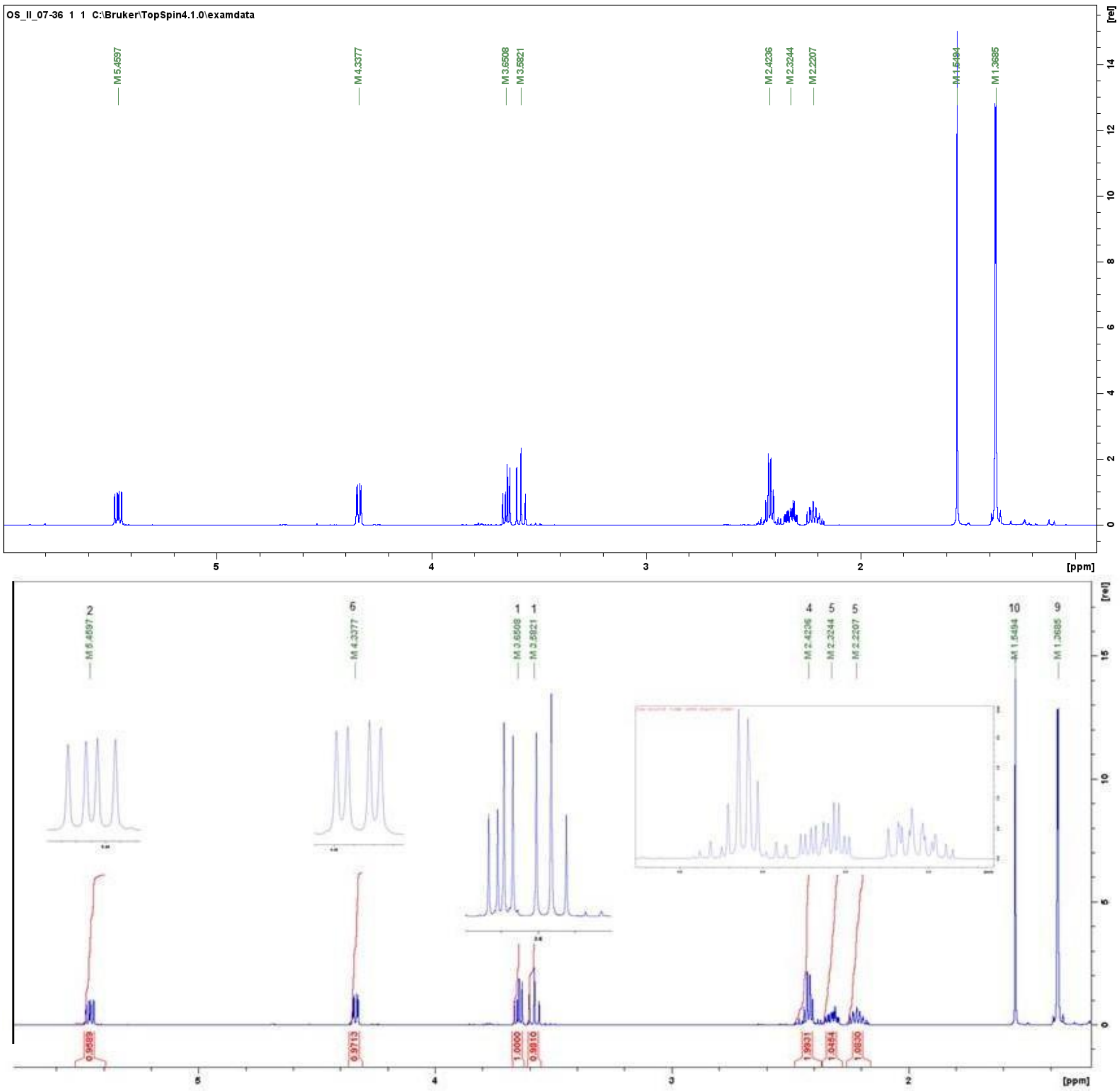

Figure S8. ^{1}H NMR spectrum of ochtodene 2.

Heteronuclear Single Quantum Correlation – HSQC ($CDCl_3$, 500 MHz)

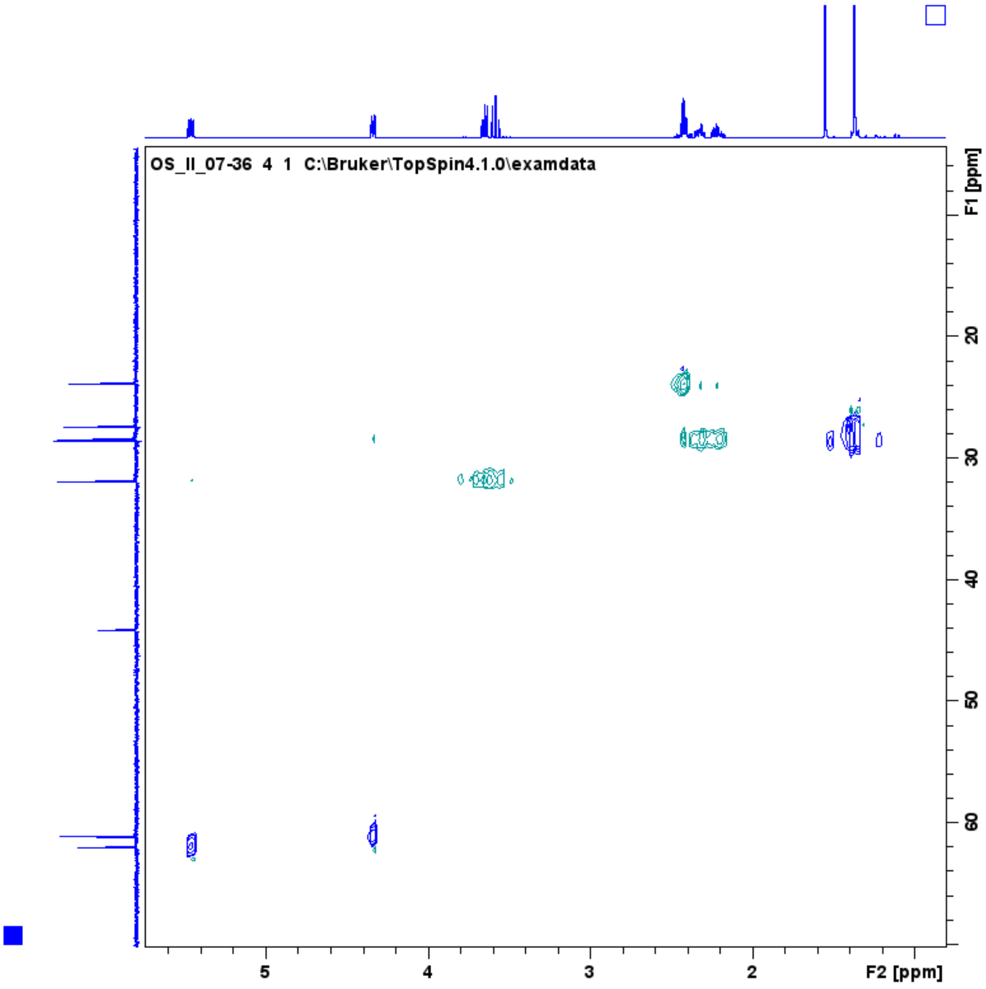

Figure S9. HSQC correlation at ochtodene 2.

Correlation Spectroscopy – COSY (CDCl3, 500 MHz)

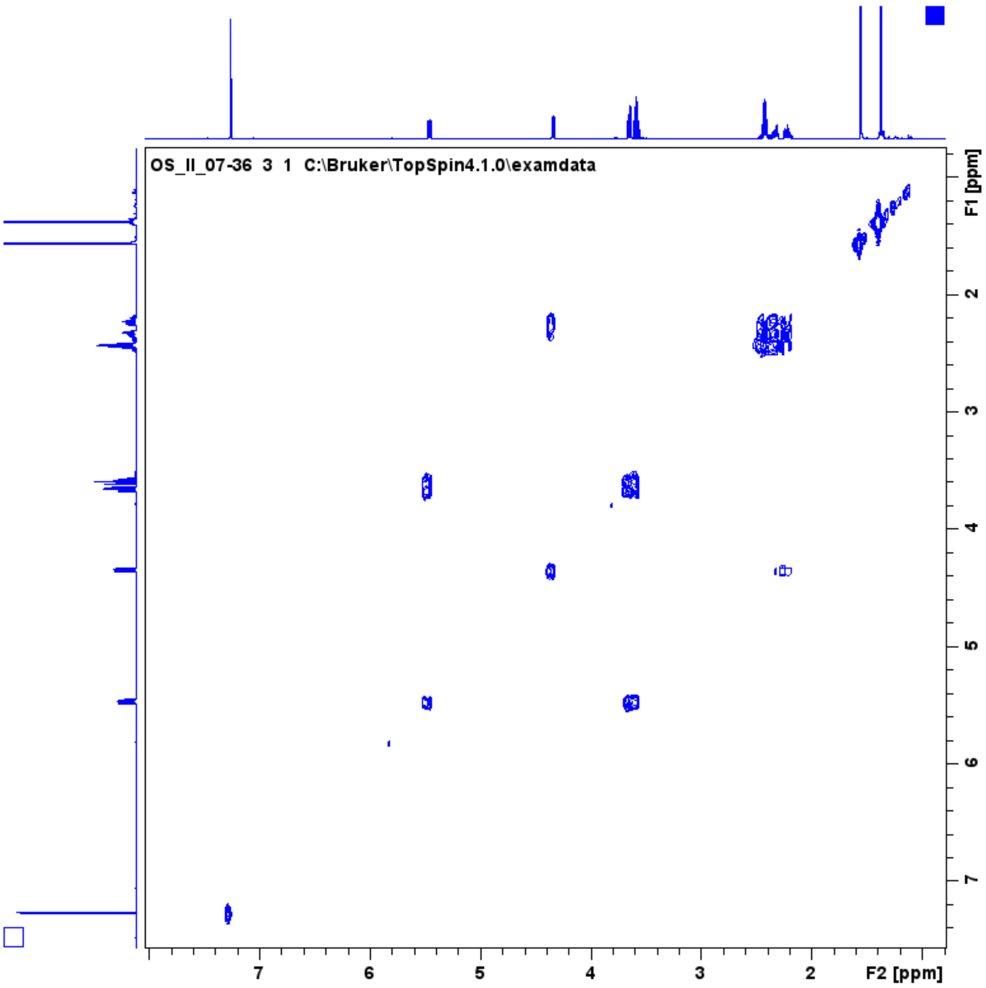

Figure S10. COSY correlation in ochtodene 2.

Heteronuclear Multiple Bond Correlation – HMBC (CDCl3, 500 MHz)

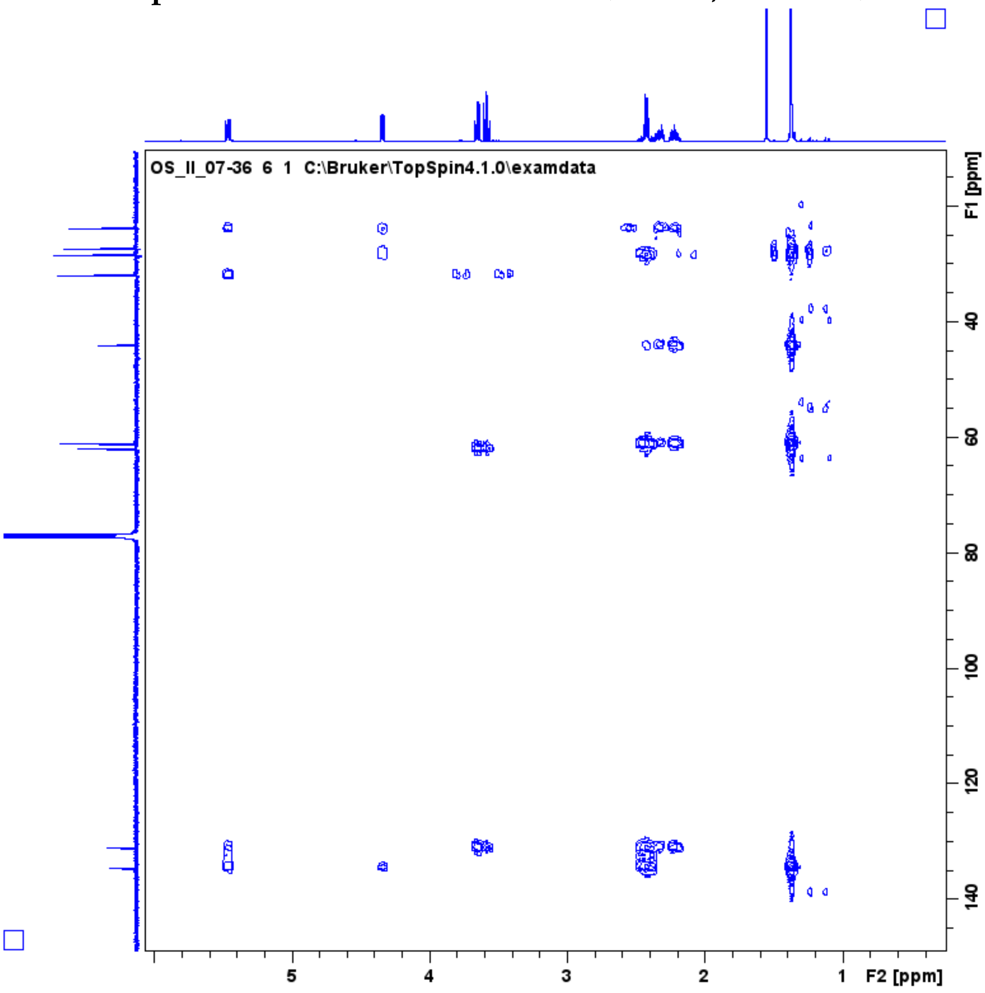

Figure S11. HMBC correlation at ochtodene 2.

Mass Spectrometry - MS.

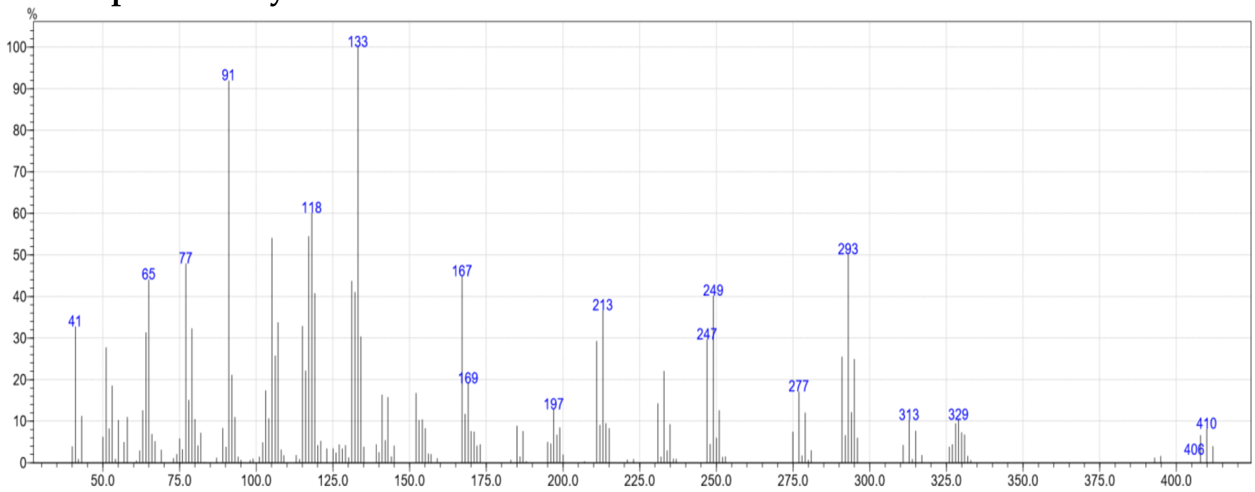

Figure S12. Mass spectrum of ochtodene 2.

Ochtodene 3 ($C_{10}H_{15}Br_2Cl$):

^{13}Carbom NMR spectrum (CDCl3, 500 MHz)

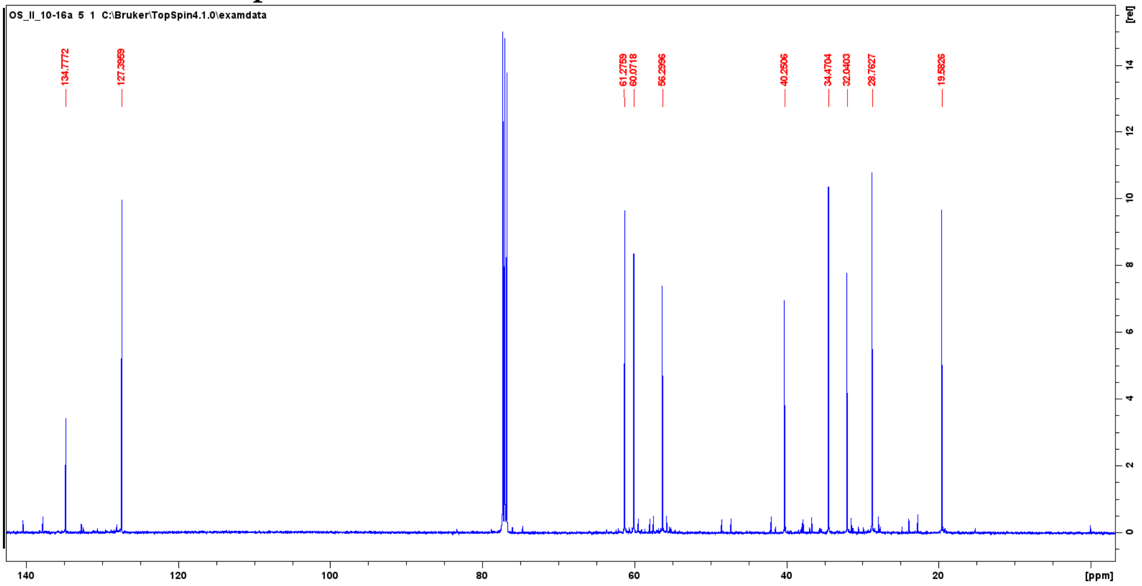

Figure S13. ^{13}C NMR spectrum of ochtodene 3.

^{1}H NMR spectrum ($CDCl_3$, 500 MHz)

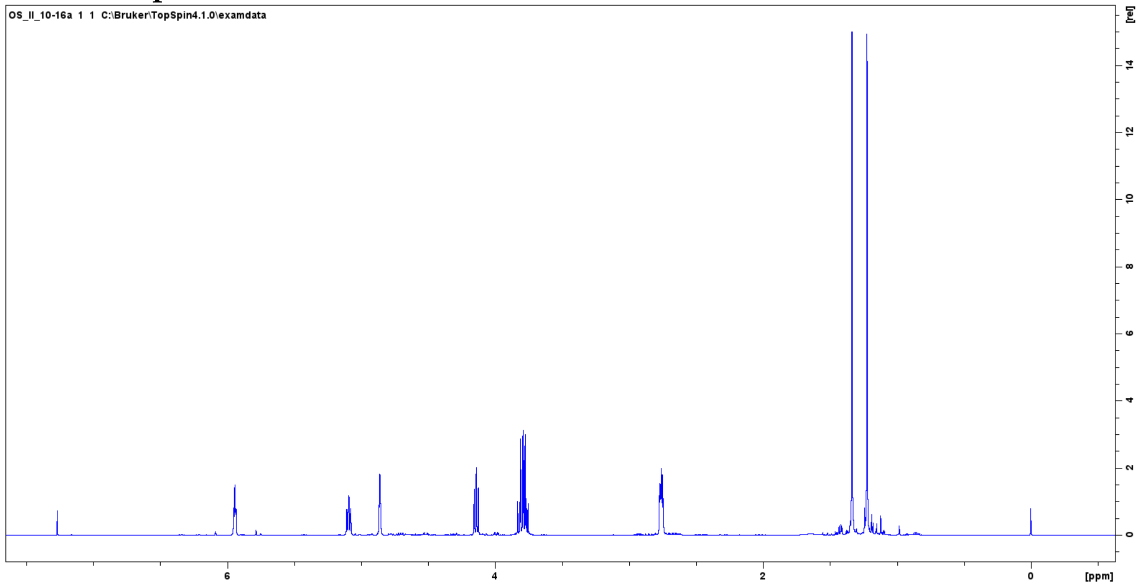

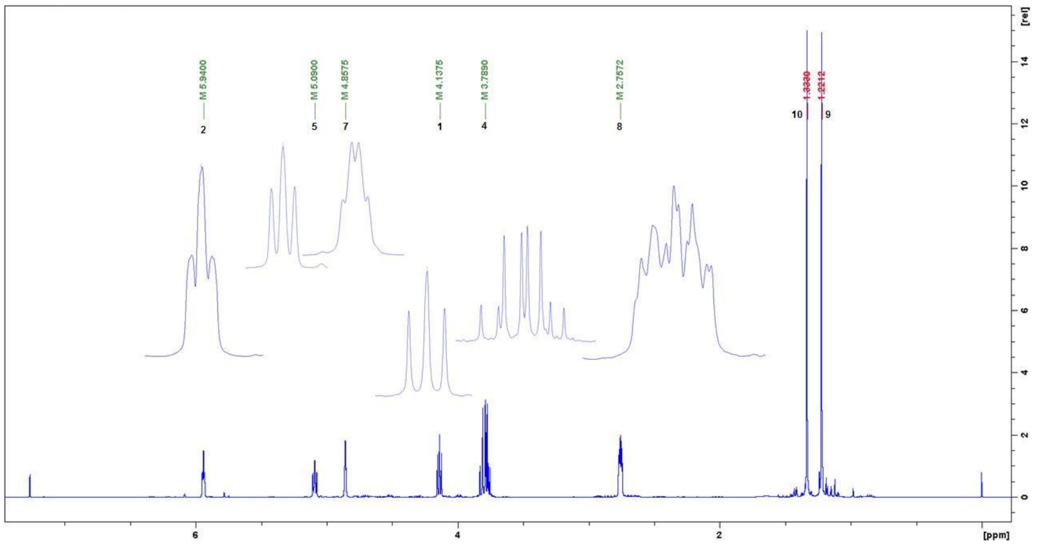

Figure S14. ^{1}H NMR spectrum of ochtodene 3.

Heteronuclear Single Quantum Correlation – HSQC ($CDCl_3$, 500 MHz)

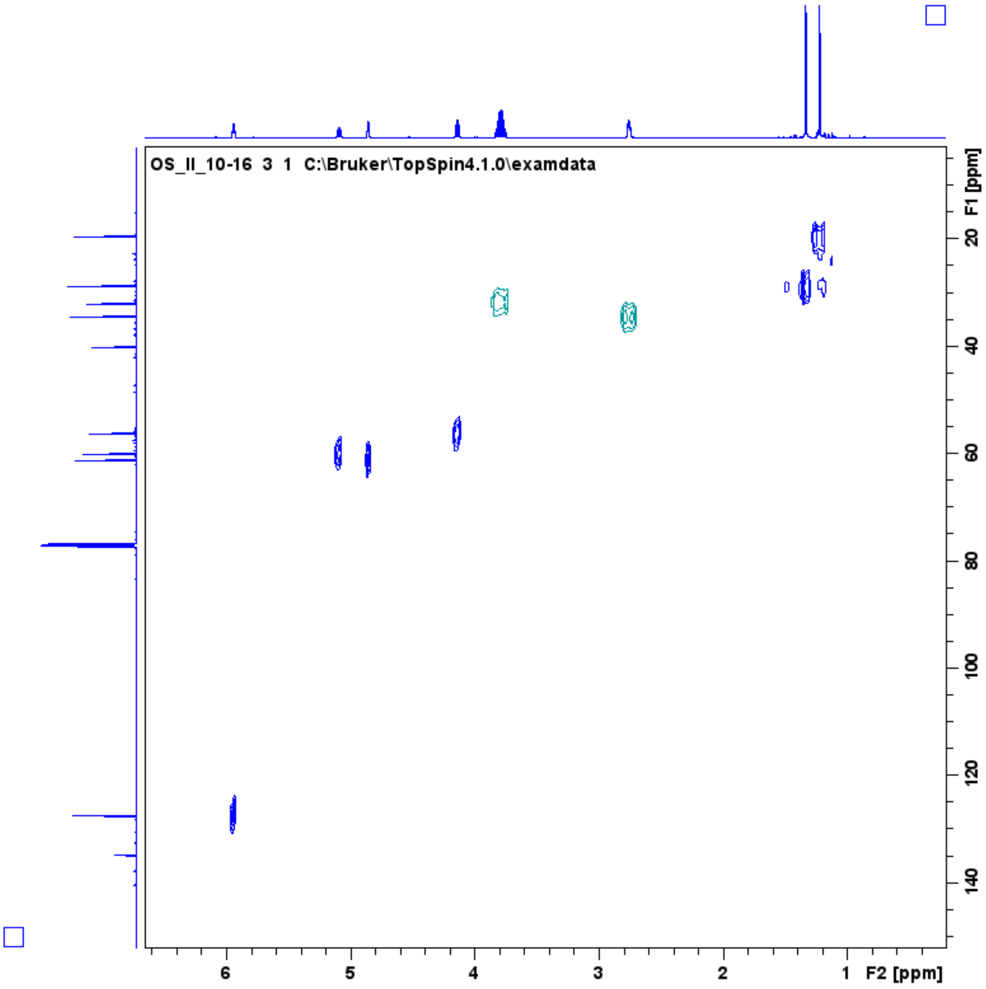

Figure S15. HSQC correlation at ochtodene 3.

Heteronuclear Multiple Bond Correlation – HMBC (CDCl3, 500 MHz)

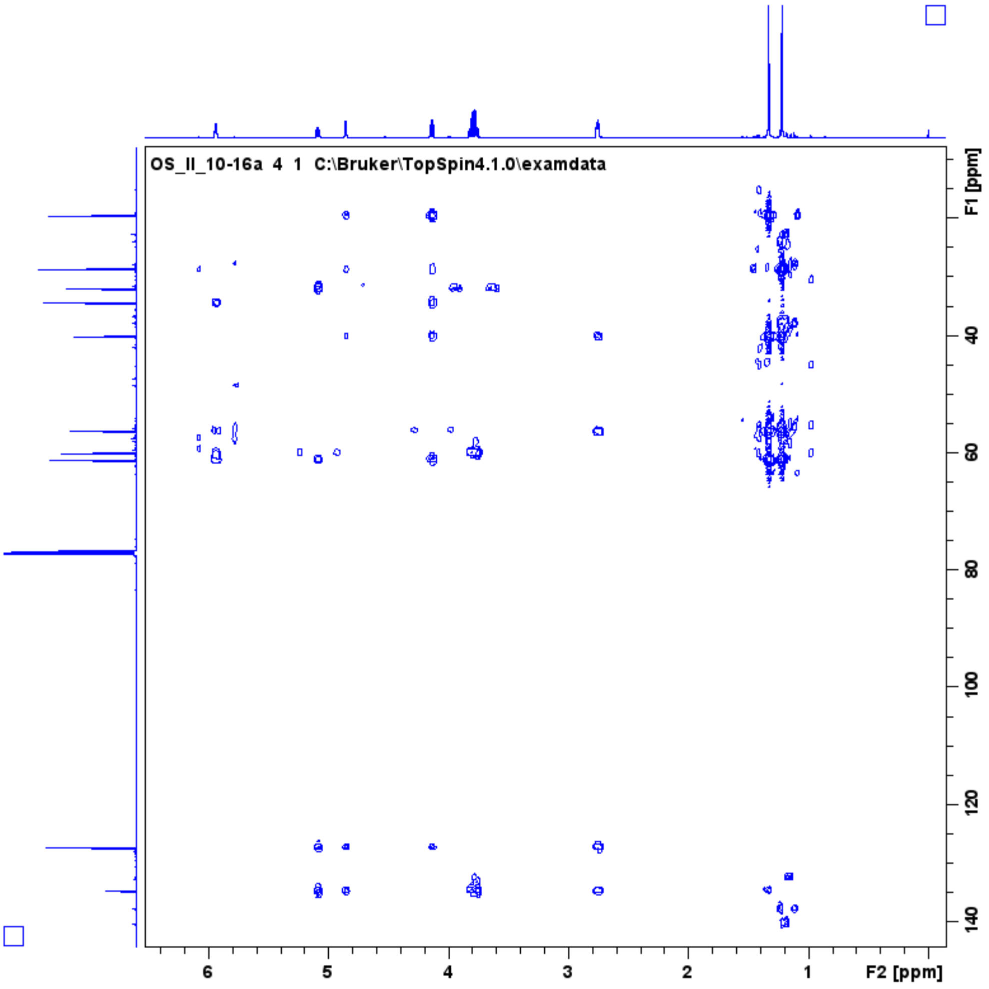

Figure S16. HMBC correlation at ochtodene 3.

Correlation Spectroscopy – COSY (CDCl3, 500 MHz)

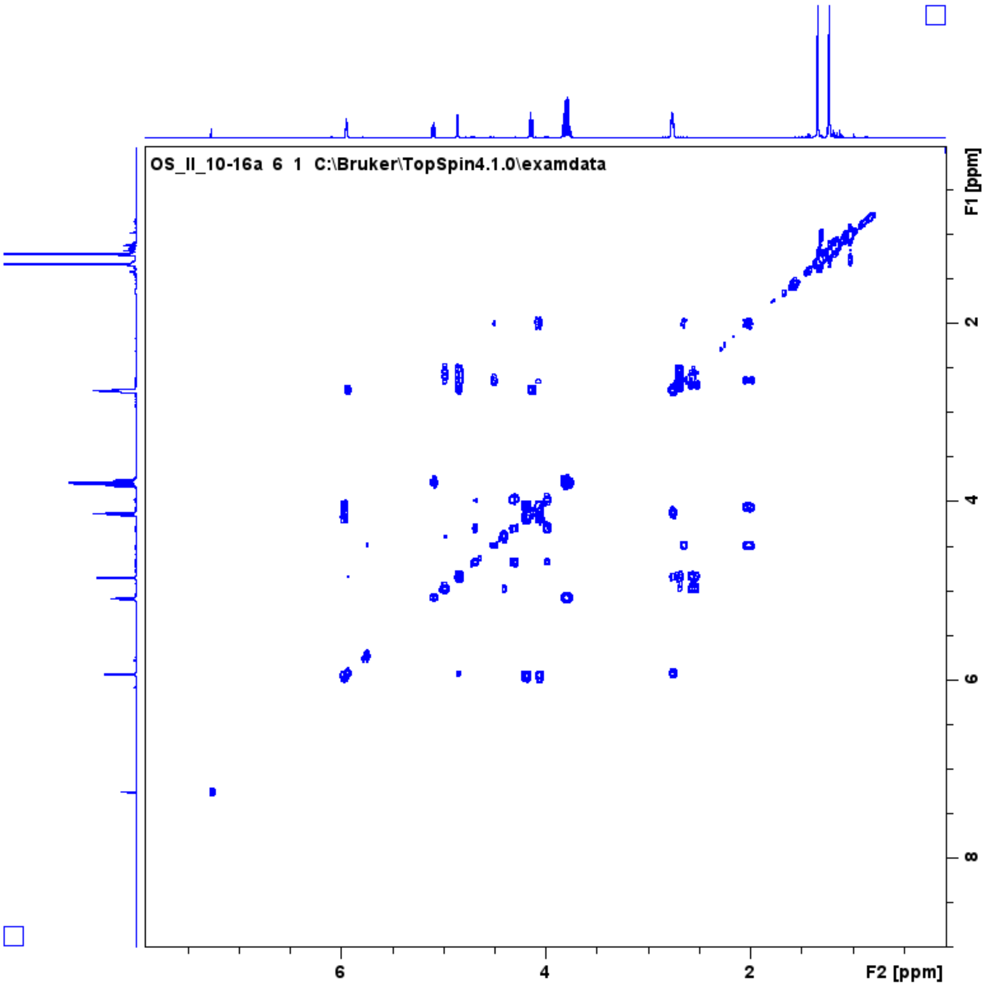

Figure S17. COSY correlation in ochtodene 3.

Mass Spectrometry – MS

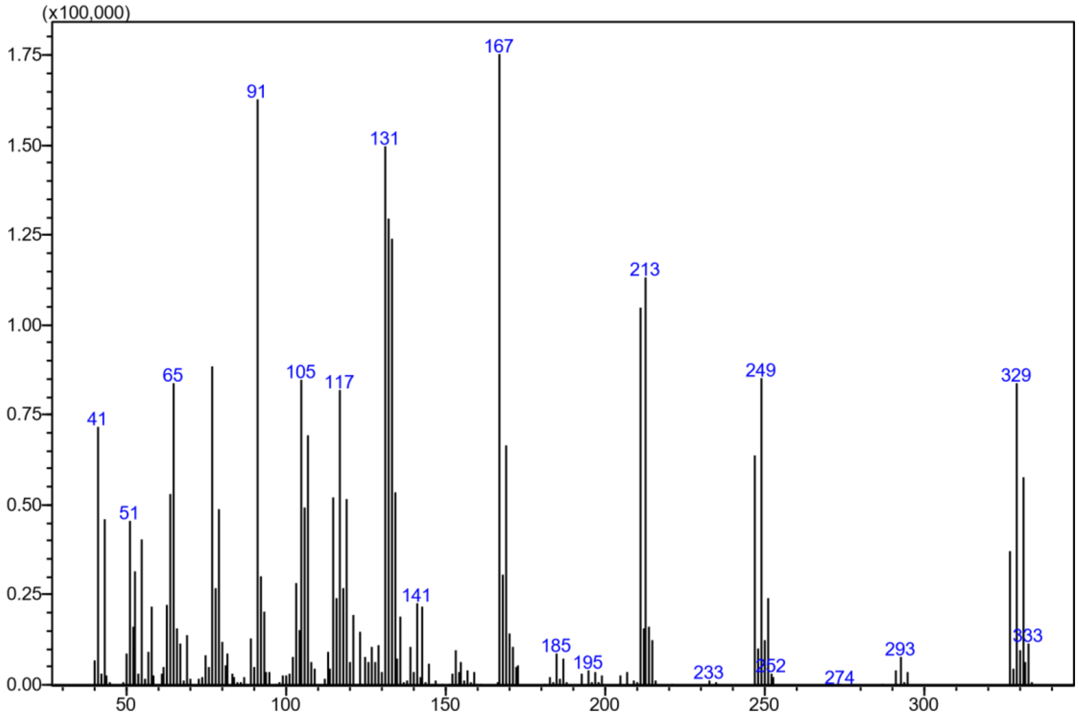

Figure S18. Mass spectrum of ochtodene 3.
